# Supplementary material for: Impact of dietary interventions on pre-diabetic oral and gut microbiome, metabolites and cytokines
Source: Nat Commun. 2023 Sep 4;14:5384. doi: 10.1038/s41467-023-41042-x (PMC10477304; doi:10.1038/s41467-023-41042-x)
Supplement: Supplementary file 1 — Supplementary Information [file 41467_2023_41042_MOESM1_ESM.pdf]

Impact of Dietary Interventions on Pre-diabetic Oral and Gut Microbiome, Metabolites and Cytokines

Supplementary Information

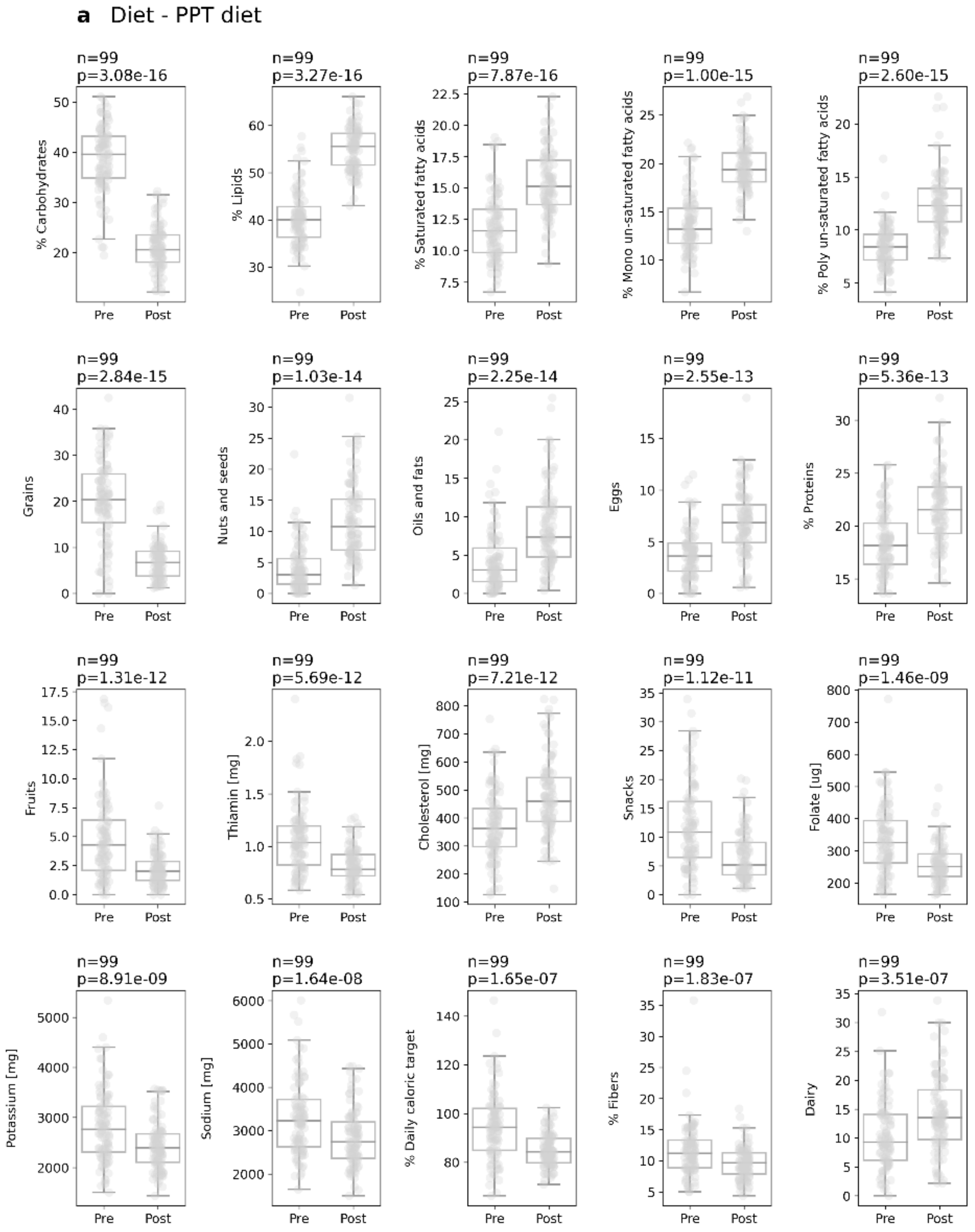

a Diet - PPT diet
n=99 p=3.08e-16
% Carbohydrates
n=99 p=3.27e-16
% Lipids
n=99 p=7.87e-16
% Saturated fatty acids
n=99 p=1.00e-15
% Mono un-saturated fatty acids
n=99 p=2.60e-15
% Poly un-saturated fatty acids
n=99 p=2.84e-15
Grains
n=99 p=1.03e-14
Nuts and seeds
n=99 p=2.25e-14
Oils and fats
n=99 p=2.55e-13
Eggs
n=99 p=5.36e-13
% Proteins
n=99 p=1.31e-12
Fruits
n=99 p=5.69e-12
Thiamin [mg]
n=99 p=7.21e-12
Cholesterol [mg]
n=99 p=1.12e-11
Snacks
n=99 p=1.46e-09
Folate [ug]
n=99 p=8.91e-09
Potassium [mg]
n=99 p=1.64e-08
Sodium [mg]
n=99 p=1.65e-07
% Daily caloric target
n=99 p=1.83e-07
% Fibers
n=99 p=3.51e-07
Dairy
Pre
Post

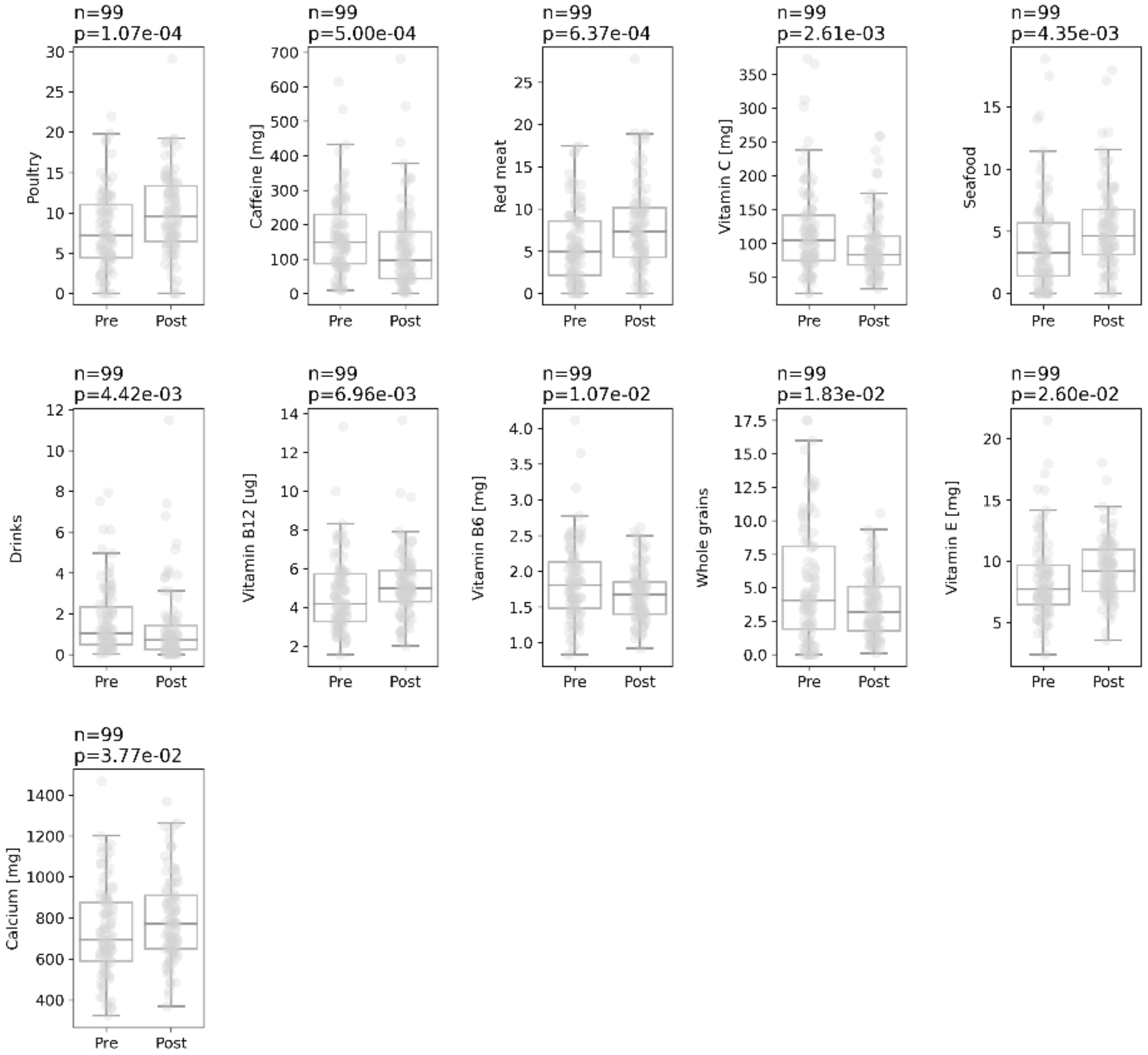
n=99
p=1.07e-04
Poultry
n=99
p=5.00e-04
Caffeine [mg]
n=99
p=6.37e-04
Red meat
n=99
p=2.61e-03
Vitamin C [mg]
n=99
p=4.35e-03
Seafood
n=99
p=4.42e-03
Drinks
n=99
p=6.96e-03
Vitamin B12 [ug]
n=99
p=1.07e-02
Vitamin B6 [mg]
n=99
p=1.83e-02
Whole grains
n=99
p=2.60e-02
Vitamin E [mg]
n=99
p=3.77e-02
Calcium [mg]
Pre
Post

b Diet - MED diet

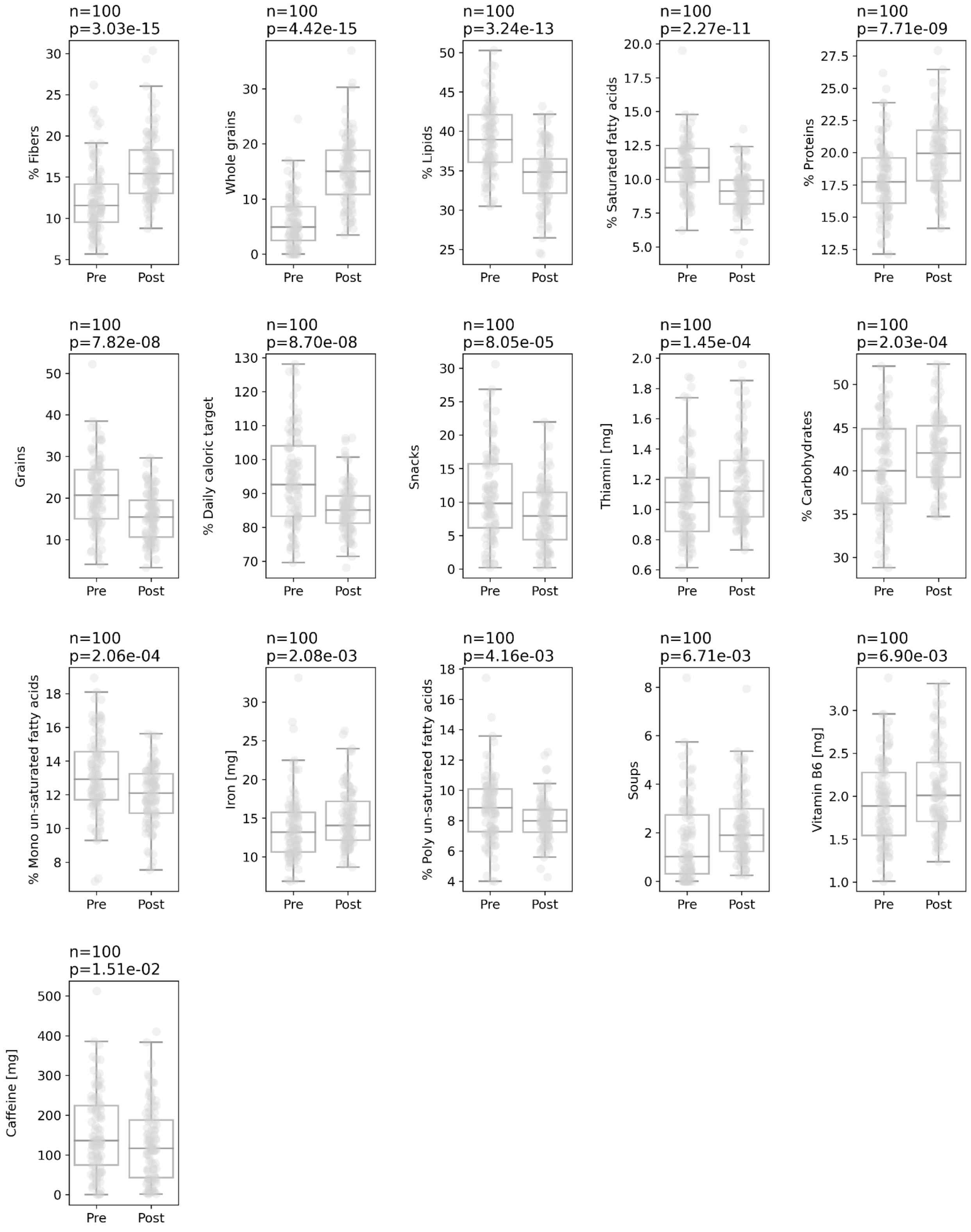

c Gut species - PPT diet

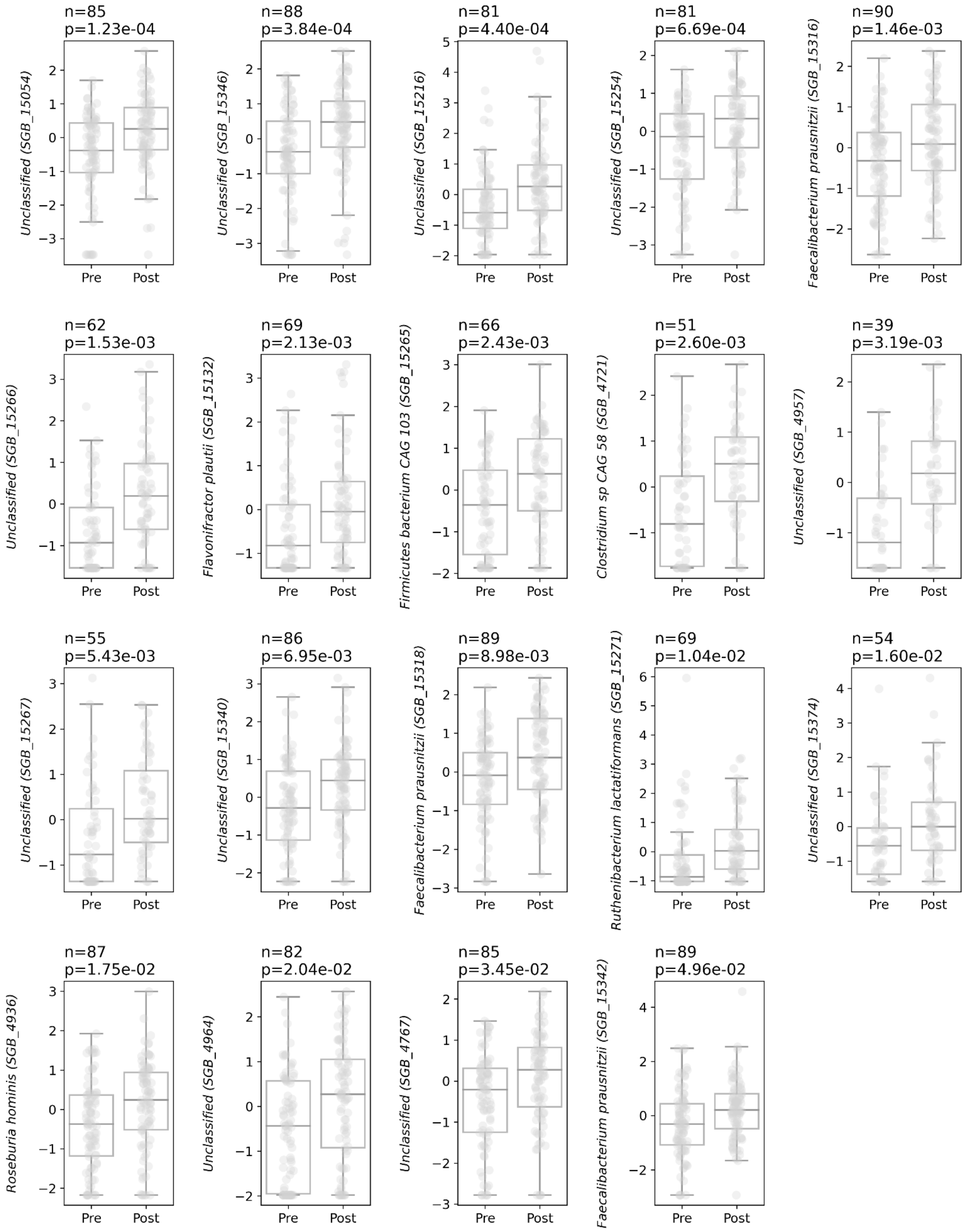

d Gut species - MED diet

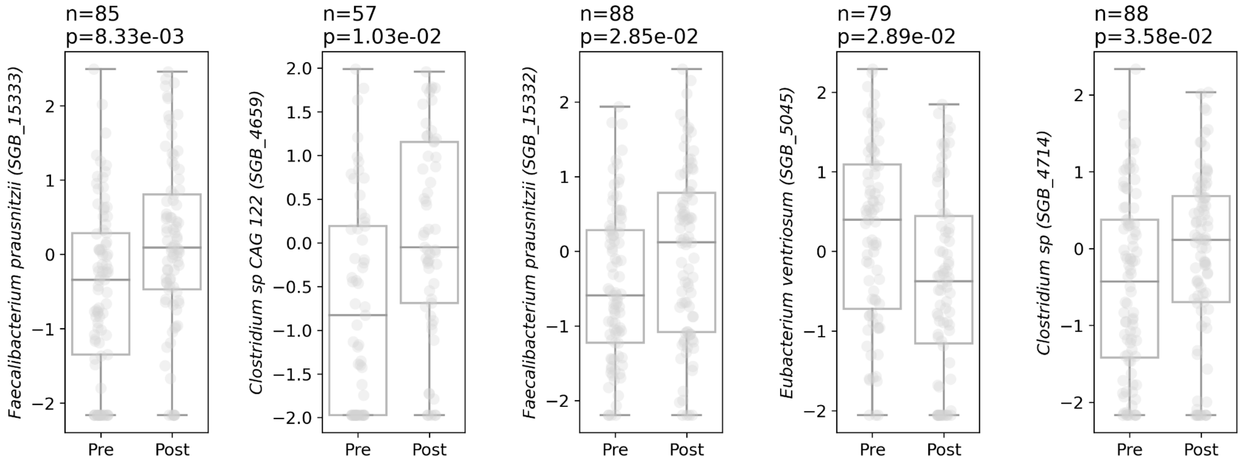

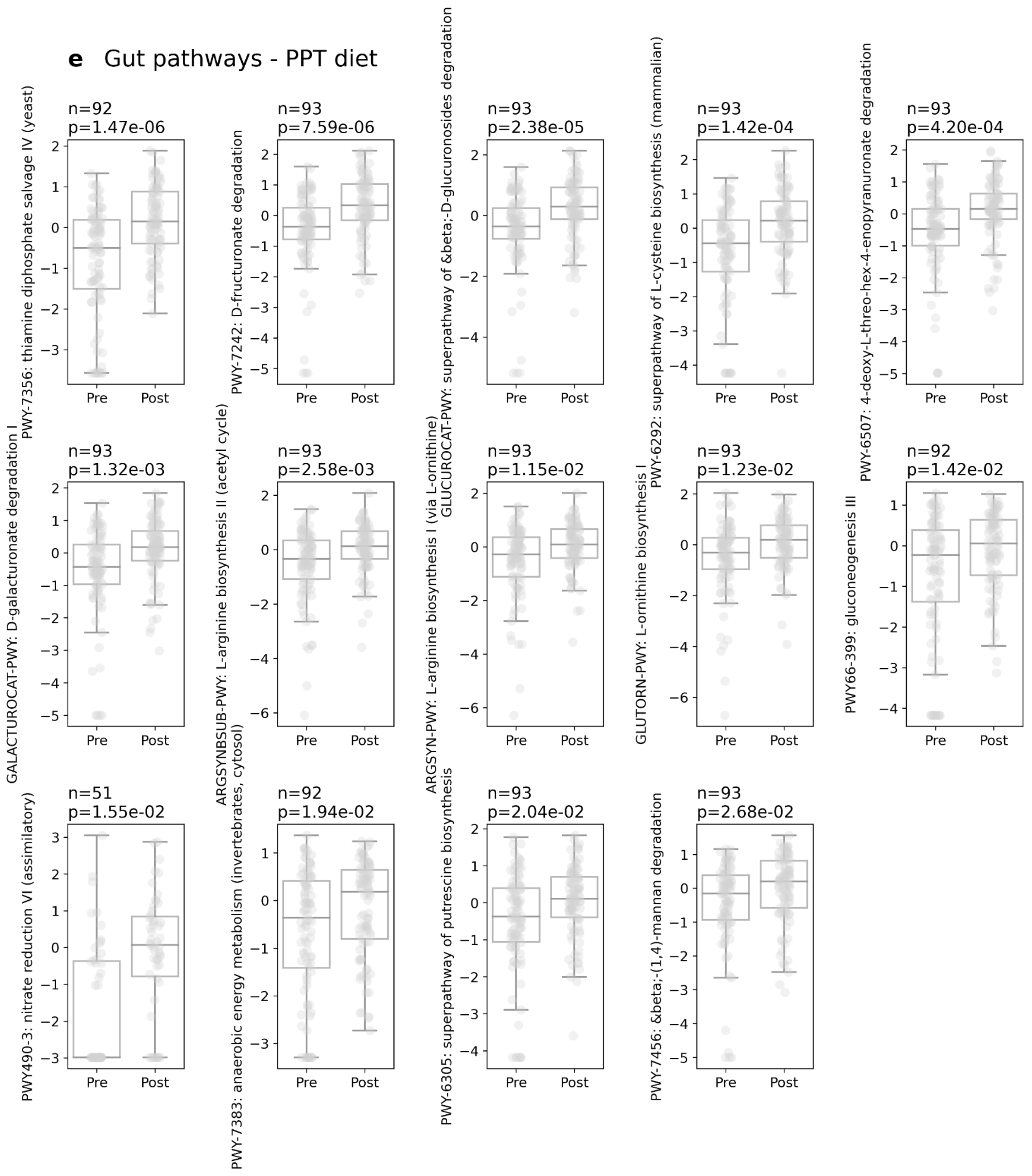
e Gut pathways - PPT diet
n=92
p=1.47e-06
PWY-7356: thiamine diphosphate salvage IV (yeast)
n=93
p=7.59e-06
PWY-7242: D-fructuronate degradation
n=93
p=2.38e-05
GLUCUROCAT-PWY: superpathway of β-D-glucuronosides degradation
n=93
p=1.42e-04
PWY-6292: superpathway of L-cysteine biosynthesis (mammalian)
n=93
p=4.20e-04
PWY-6507: 4-deoxy-L-threo-hex-4-enopyranuronate degradation
n=93
p=1.32e-03
GALACTUROCAT-PWY: D-galacturonate degradation I
n=93
p=2.58e-03
ARGSYNBSUB-PWY: L-arginine biosynthesis II (acetyl cycle)
n=93
p=1.15e-02
ARGSYN-PWY: L-arginine biosynthesis I (via L-ornithine)
n=93
p=1.23e-02
GLUTORN-PWY: L-ornithine biosynthesis I
n=92
p=1.42e-02
PWY66-399: gluconeogenesis III
n=51
p=1.55e-02
PWY490-3: nitrate reduction VI (assimilatory)
n=92
p=1.94e-02
PWY-7383: anaerobic energy metabolism (invertebrates, cytosol)
n=93
p=2.04e-02
PWY-6305: superpathway of putrescine biosynthesis
n=93
p=2.68e-02
PWY-7456: β-(1,4)-mannan degradation
Pre
Post

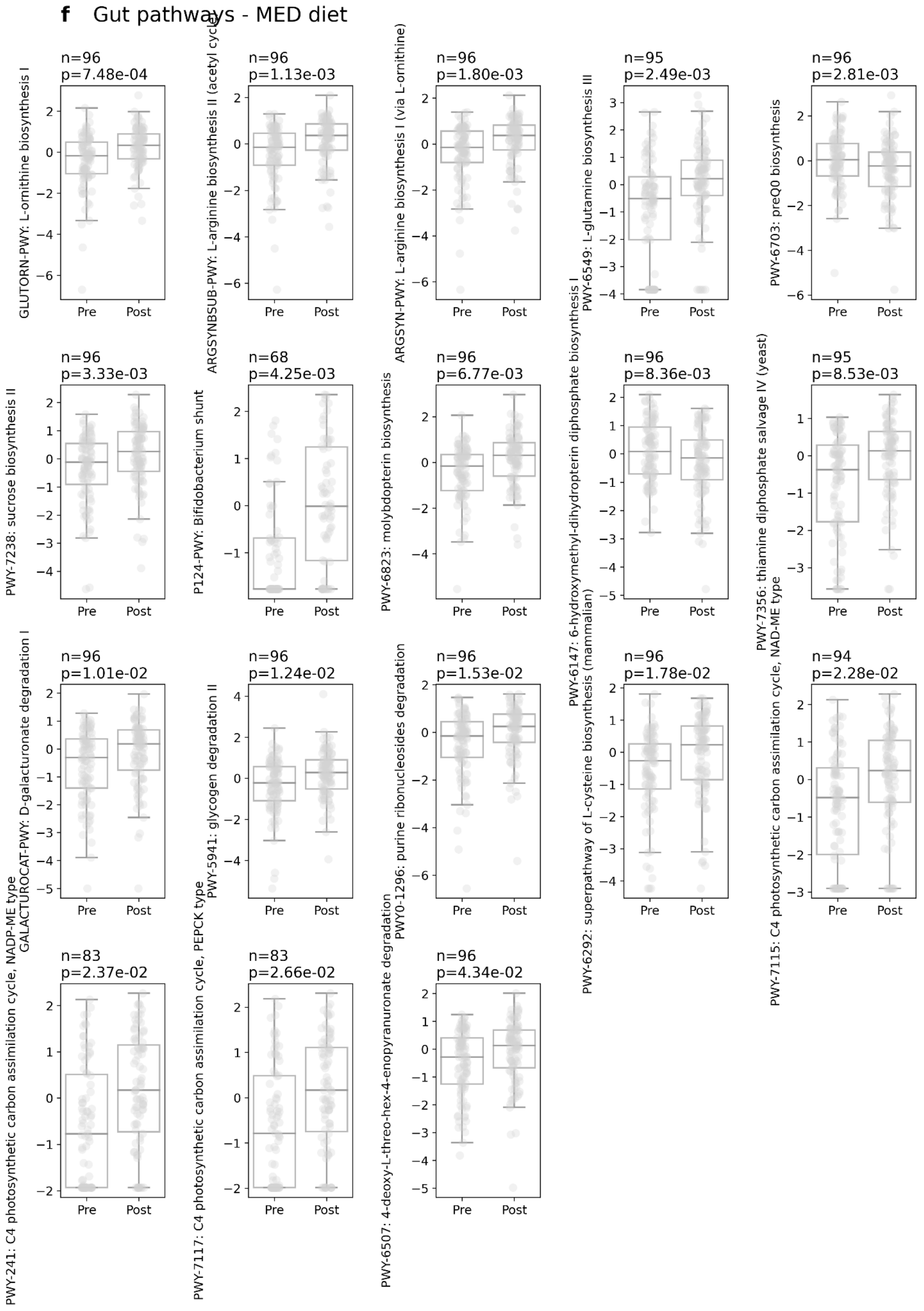

f Gut pathways - MED diet
n=96 p=7.48e-04
GLUTORN-PWY: L-ornithine biosynthesis I
n=96 p=1.13e-03
ARGSYNBSUB-PWY: L-arginine biosynthesis II (acetyl cycle)
n=96 p=1.80e-03
ARGSYN-PWY: L-arginine biosynthesis I (via L-ornithine)
n=95 p=2.49e-03
PWY-6549: L-glutamine biosynthesis III
n=96 p=2.81e-03
PWY-6703: preQ0 biosynthesis
n=96 p=3.33e-03
PWY-7238: sucrose biosynthesis II
n=68 p=4.25e-03
P124-PWY: Bifidobacterium shunt
n=96 p=6.77e-03
PWY-6823: molybdopterin biosynthesis
n=96 p=8.36e-03
PWY-6147: 6-hydroxymethyl-dihydropterin diphosphate biosynthesis I
n=95 p=8.53e-03
PWY-7356: thiamine diphosphate salvage IV (yeast)
n=96 p=1.01e-02
GALACTUROCAT-PWY: D-galacturonate degradation I
n=96 p=1.24e-02
PWY-5941: glycogen degradation II
n=96 p=1.53e-02
PWY0-1296: purine ribonucleosides degradation
n=96 p=1.78e-02
PWY-6292: superpathway of L-cysteine biosynthesis (mammalian)
n=94 p=2.28e-02
PWY-7115: C4 photosynthetic carbon assimilation cycle, NAD-ME type
n=83 p=2.37e-02
PWY-241: C4 photosynthetic carbon assimilation cycle, NADP-ME type
n=83 p=2.66e-02
PWY-7117: C4 photosynthetic carbon assimilation cycle, PEPCK type
n=96 p=4.34e-02
PWY-6507: 4-deoxy-L-threo-hex-4-enopyranuronate degradation
Pre
Post

g Oral species - MED diet

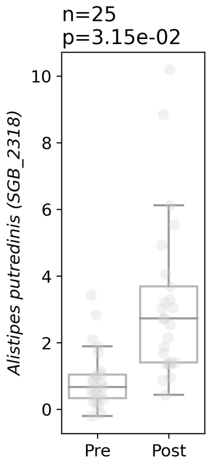

h Oral pathways - PPT diet

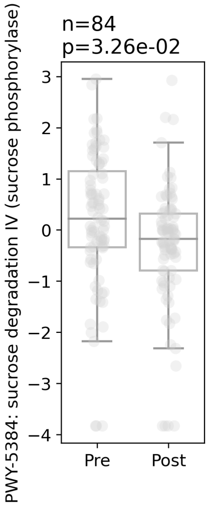

i Metabolites - PPT diet

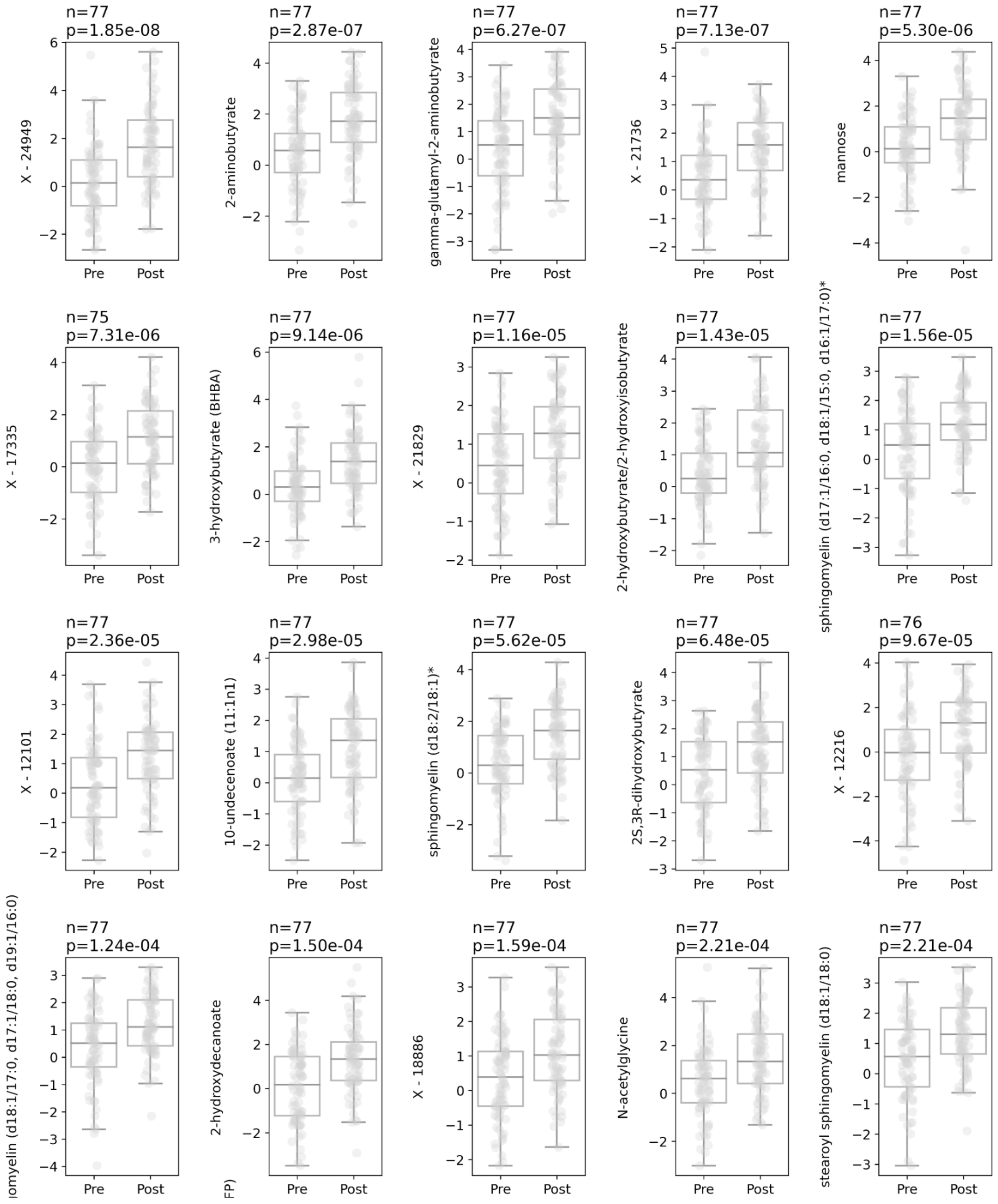

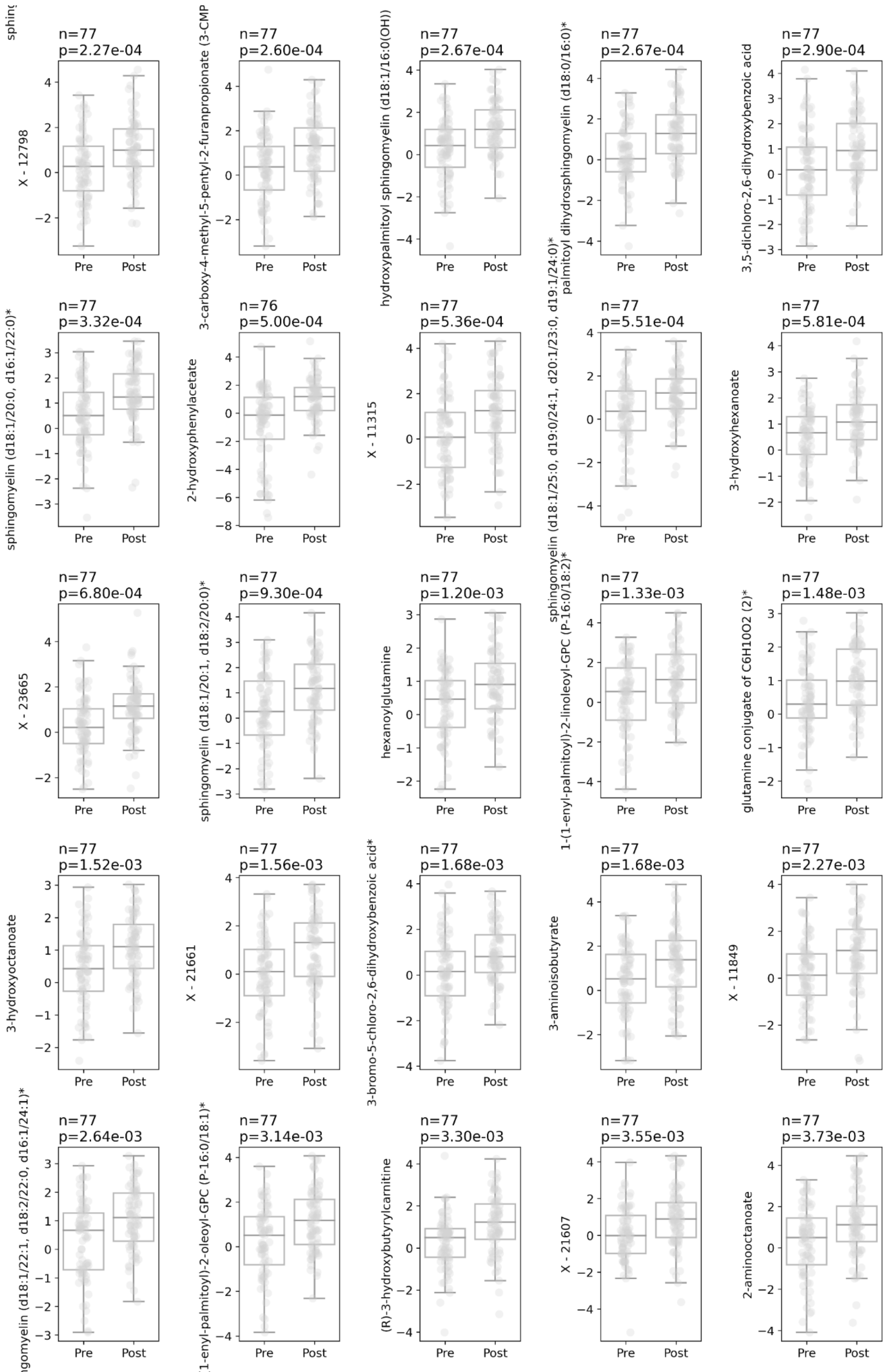

sphinç
n=77
p=2.27e-04
X - 12798
3-carboxy-4-methyl-5-pentyl-2-furanpropionate (3-CMP
n=77
p=2.60e-04
hydroxypalmitoyl sphingomyelin (d18:1/16:0(OH))
n=77
p=2.67e-04
palmitoyl dihydrosphingomyelin (d18:0/16:0)*
n=77
p=2.67e-04
3,5-dichloro-2,6-dihydroxybenzoic acid
n=77
p=2.90e-04
sphingomyelin (d18:1/20:0, d16:1/22:0)*
n=77
p=3.32e-04
2-hydroxyphenylacetate
n=76
p=5.00e-04
X - 11315
n=77
p=5.36e-04
sphingomyelin (d18:1/25:0, d19:0/24:1, d20:1/23:0, d19:1/24:0)*
n=77
p=5.51e-04
3-hydroxyhexanoate
n=77
p=5.81e-04
X - 23665
n=77
p=6.80e-04
sphingomyelin (d18:1/20:1, d18:2/20:0)*
n=77
p=9.30e-04
hexanoylglutamine
n=77
p=1.20e-03
1-(1-enyl-palmitoyl)-2-linoleoyl-GPC (P-16:0/18:2)*
n=77
p=1.33e-03
glutamine conjugate of C6H10O2 (2)*
n=77
p=1.48e-03
3-hydroxyoctanoate
n=77
p=1.52e-03
X - 21661
n=77
p=1.56e-03
3-bromo-5-chloro-2,6-dihydroxybenzoic acid*
n=77
p=1.68e-03
3-aminoisobutyrate
n=77
p=1.68e-03
X - 11849
n=77
p=2.27e-03
ngomyelin (d18:1/22:1, d18:2/22:0, d16:1/24:1)*
n=77
p=2.64e-03
1-enyl-palmitoyl)-2-oleoyl-GPC (P-16:0/18:1)*
n=77
p=3.14e-03
(R)-3-hydroxybutyrylcarnitine
n=77
p=3.30e-03
X - 21607
n=77
p=3.55e-03
2-aminooctanoate
n=77
p=3.73e-03
Pre
Post

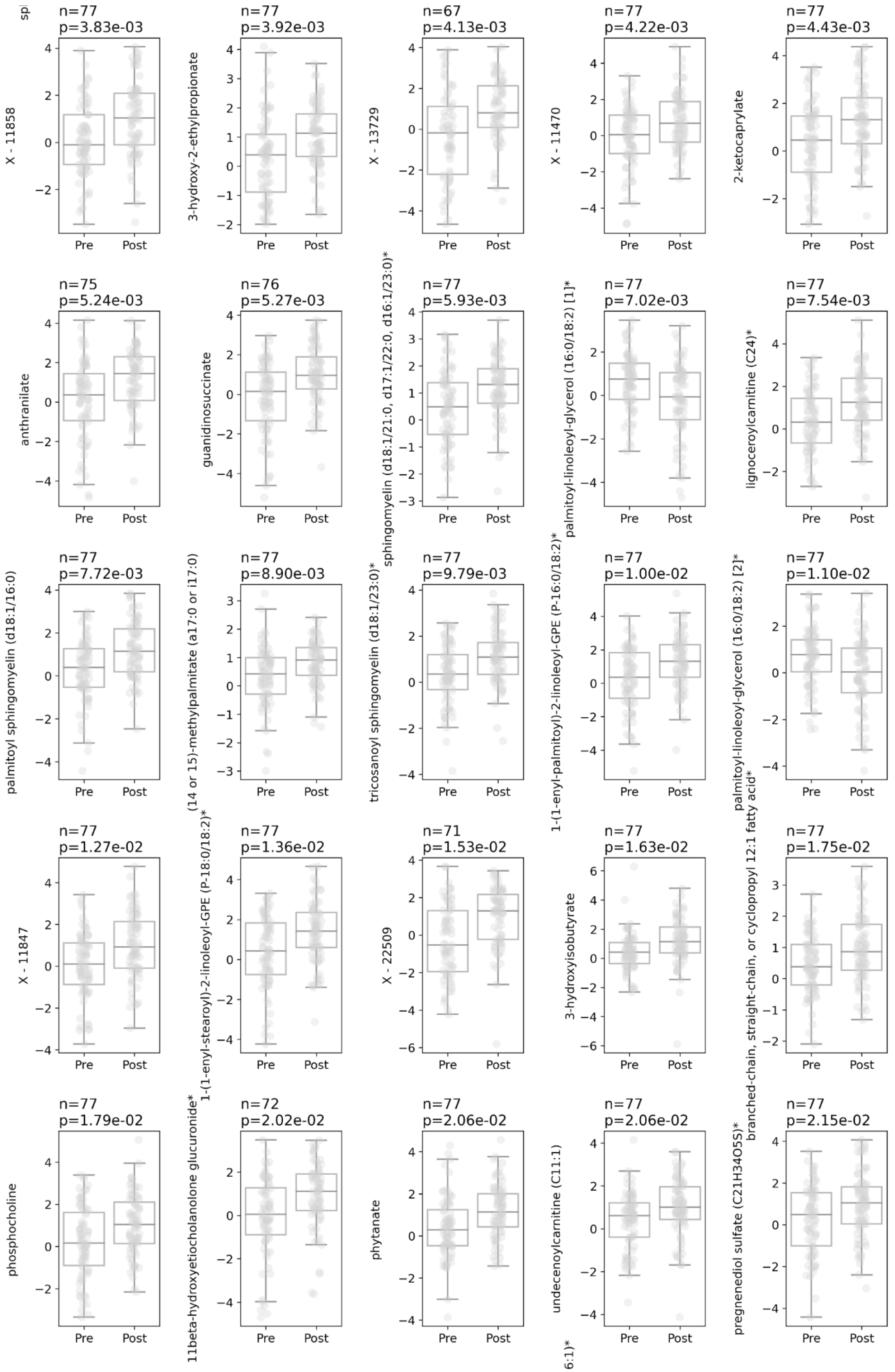

sp
X - 11858
n=77
p=3.83e-03
3-hydroxy-2-ethylpropionate
n=77
p=3.92e-03
X - 13729
n=67
p=4.13e-03
X - 11470
n=77
p=4.22e-03
2-ketocaprylate
n=77
p=4.43e-03
anthranilate
n=75
p=5.24e-03
guanidinosuccinate
n=76
p=5.27e-03
sphingomyelin (d18:1/21:0, d17:1/22:0, d16:1/23:0)*
n=77
p=5.93e-03
palmitoyl-linoleoyl-glycerol (16:0/18:2) [1]*
n=77
p=7.02e-03
lignoceroylcarnitine (C24)*
n=77
p=7.54e-03
palmitoyl sphingomyelin (d18:1/16:0)
n=77
p=7.72e-03
(14 or 15)-methylpalmitate (a17:0 or i17:0)
n=77
p=8.90e-03
tricosanoyl sphingomyelin (d18:1/23:0)*
n=77
p=9.79e-03
1-(1-enyl-palmitoyl)-2-linoleoyl-GPE (P-16:0/18:2)*
n=77
p=1.00e-02
palmitoyl-linoleoyl-glycerol (16:0/18:2) [2]*
n=77
p=1.10e-02
X - 11847
n=77
p=1.27e-02
1-(1-enyl-stearoyl)-2-linoleoyl-GPE (P-18:0/18:2)*
n=77
p=1.36e-02
X - 22509
n=71
p=1.53e-02
3-hydroxyisobutyrate
n=77
p=1.63e-02
branched-chain, straight-chain, or cyclopropyl 12:1 fatty acid*
n=77
p=1.75e-02
phosphocholine
n=77
p=1.79e-02
11beta-hydroxyetiocholanolone glucuronide*
n=72
p=2.02e-02
phytanate
n=77
p=2.06e-02
undecenoylcarnitine (C11:1)
n=77
p=2.06e-02
pregnenediol sulfate (C21H34O5S)*
n=77
p=2.15e-02
Pre
Post
6:1)*

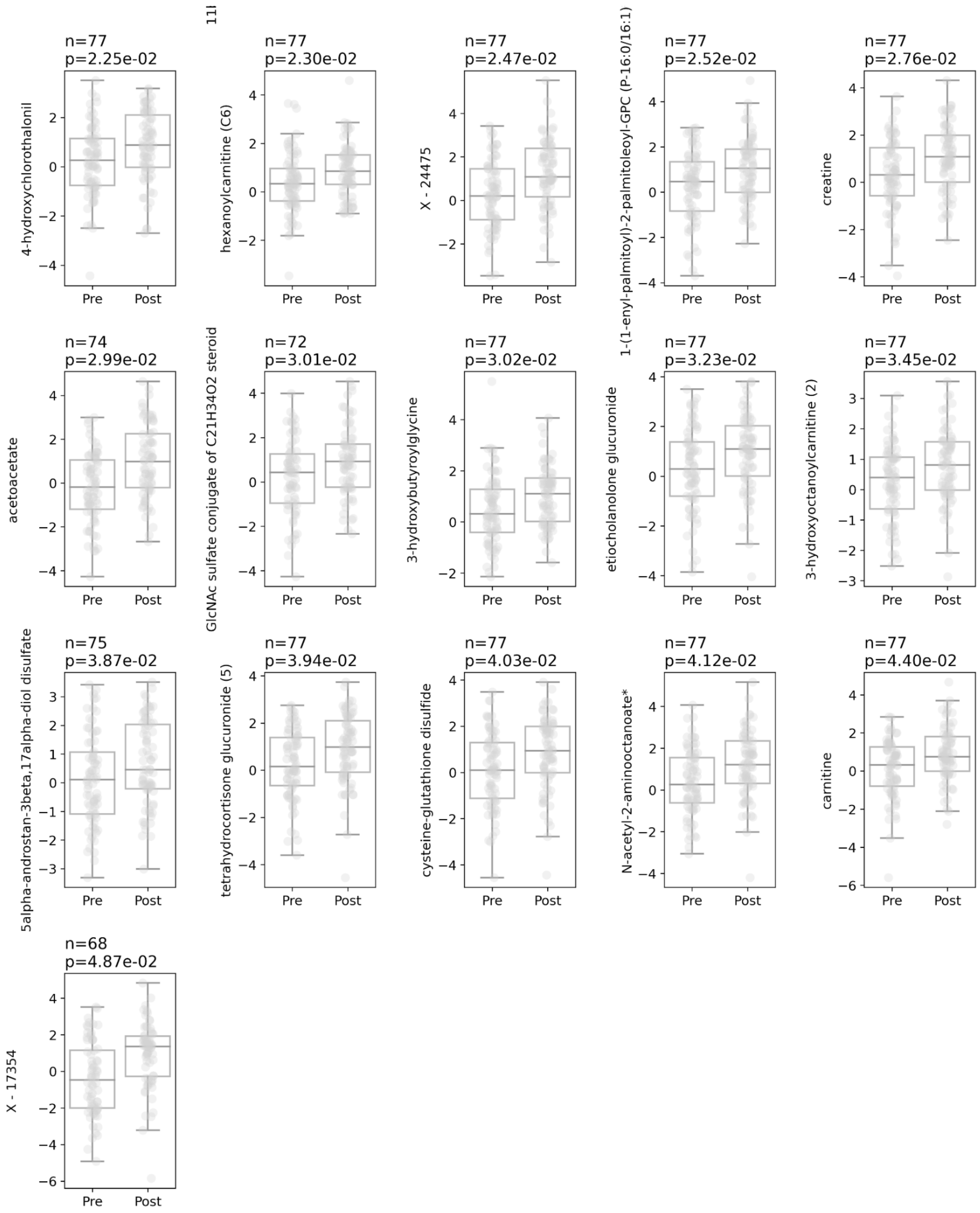
11I
n=77
p=2.25e-02
4-hydroxychlorothalonil
n=77
p=2.30e-02
hexanoylcarnitine (C6)
n=77
p=2.47e-02
X - 24475
1-(1-enyl-palmitoyl)-2-palmitoleoyl-GPC (P-16:0/16:1)
n=77
p=2.52e-02
n=77
p=2.76e-02
creatine
n=74
p=2.99e-02
acetoacetate
GlcNAc sulfate conjugate of C21H34O2 steroid
n=72
p=3.01e-02
n=77
p=3.02e-02
3-hydroxybutyroylglycine
n=77
p=3.23e-02
etiocholanolone glucuronide
n=77
p=3.45e-02
3-hydroxyoctanoylcarnitine (2)
n=75
p=3.87e-02
5alpha-androstan-3beta,17alpha-diol disulfate
n=77
p=3.94e-02
tetrahydrocortisone glucuronide (5)
n=77
p=4.03e-02
cysteine-glutathione disulfide
n=77
p=4.12e-02
N-acetyl-2-aminooctanoate*
n=77
p=4.40e-02
carnitine
n=68
p=4.87e-02
X - 17354
Pre
Post

j Metabolites - MED diet

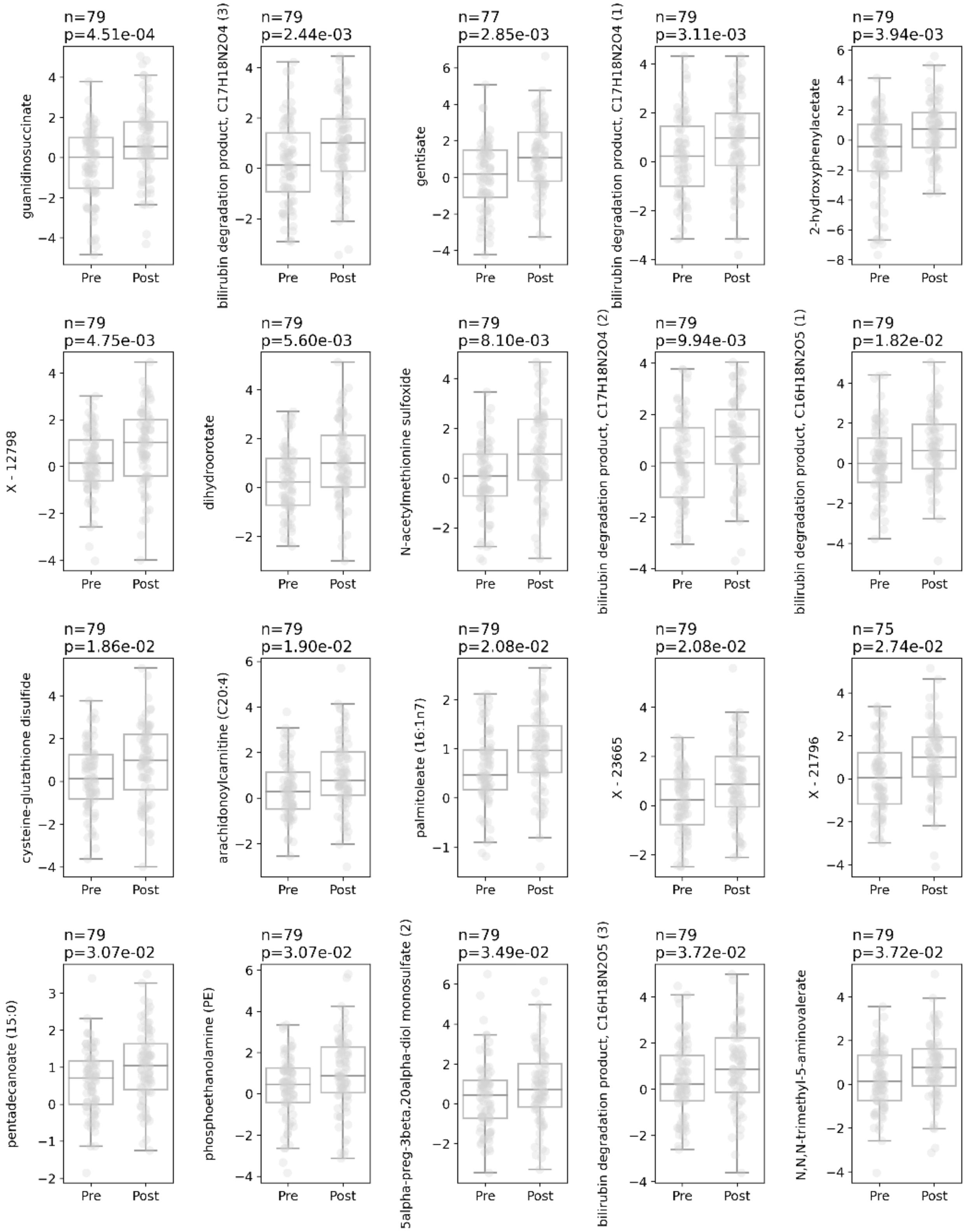

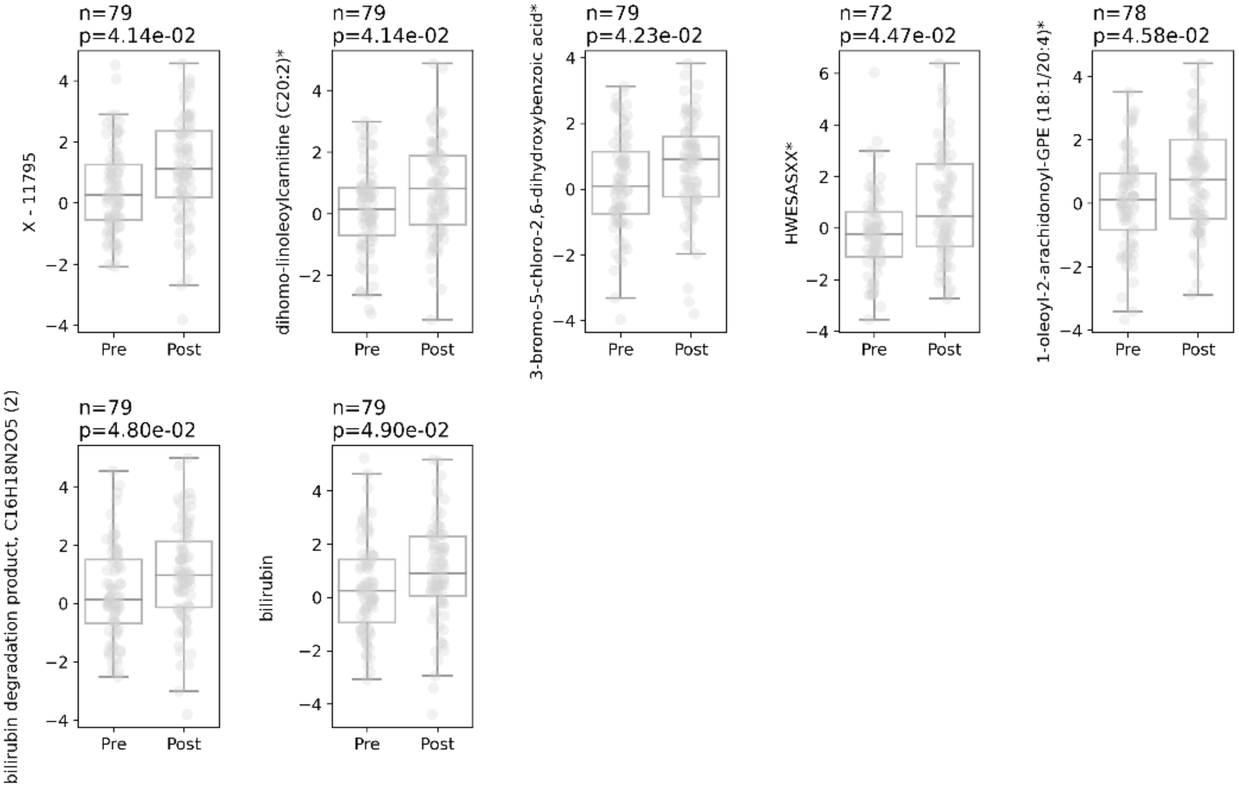
n=79
p=4.14e-02
X - 11795
n=79
p=4.14e-02
dihomo-linoleoylcarnitine (C20:2)*
n=79
p=4.23e-02
3-bromo-5-chloro-2,6-dihydroxybenzoic acid*
n=72
p=4.47e-02
HWESASXX*
n=78
p=4.58e-02
1-oleoyl-2-arachidonoyl-GPE (18:1/20:4)*
n=79
p=4.80e-02
bilirubin degradation product, C16H18N2O5 (2)
n=79
p=4.90e-02
bilirubin
Pre
Post

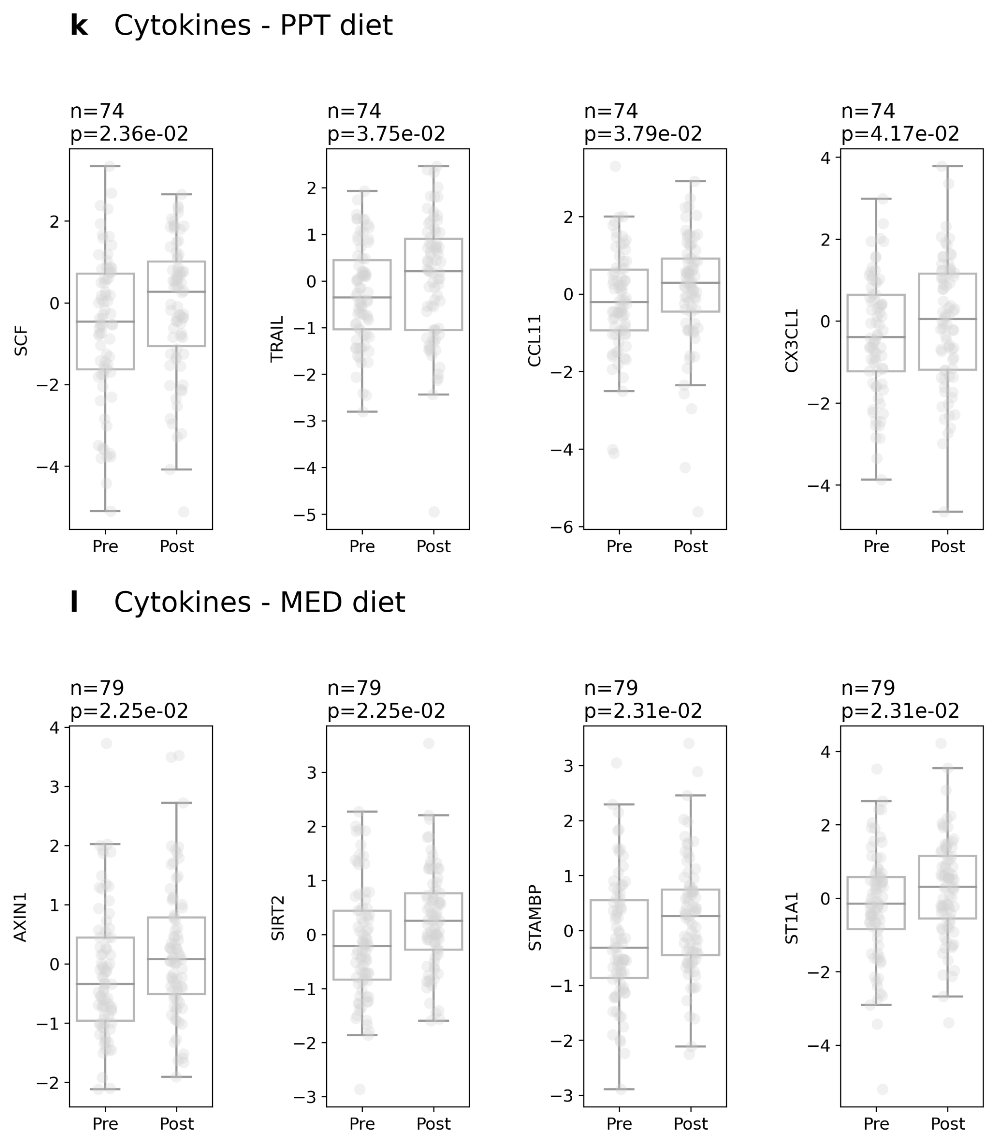

Figure S1. The PPT diet had bigger effect on the microbiome and metabolites than the MED diet

Features at baseline versus the end of the intervention that significantly changed (Bonferroni corrected $p<0.05$, two-sided Wilcoxon paired signed-rank test or false discovery rate correction solely in the cytokines). Units are standard deviations over the log10 transformed data (Methods). p - p-value, n - number of participants compared. Boxes show the quartiles of the data (0.25, 0.50, 0.75) while the whiskers extend to 1.5 of the inter quartile range, points beyond the whiskers are considered to be outliers. The title specifies the type of features (diet, microbial species, microbial pathways, metabolites or cytokines) and the diet group ("PPT" or "MED" diet). There was no significant difference between the two diet groups at baseline in any of the 2,803 molecular features tested (Bonferroni corrected $p>0.05$, two-sided Mann-Whitney U test). Source data are provided as a Source Data file.

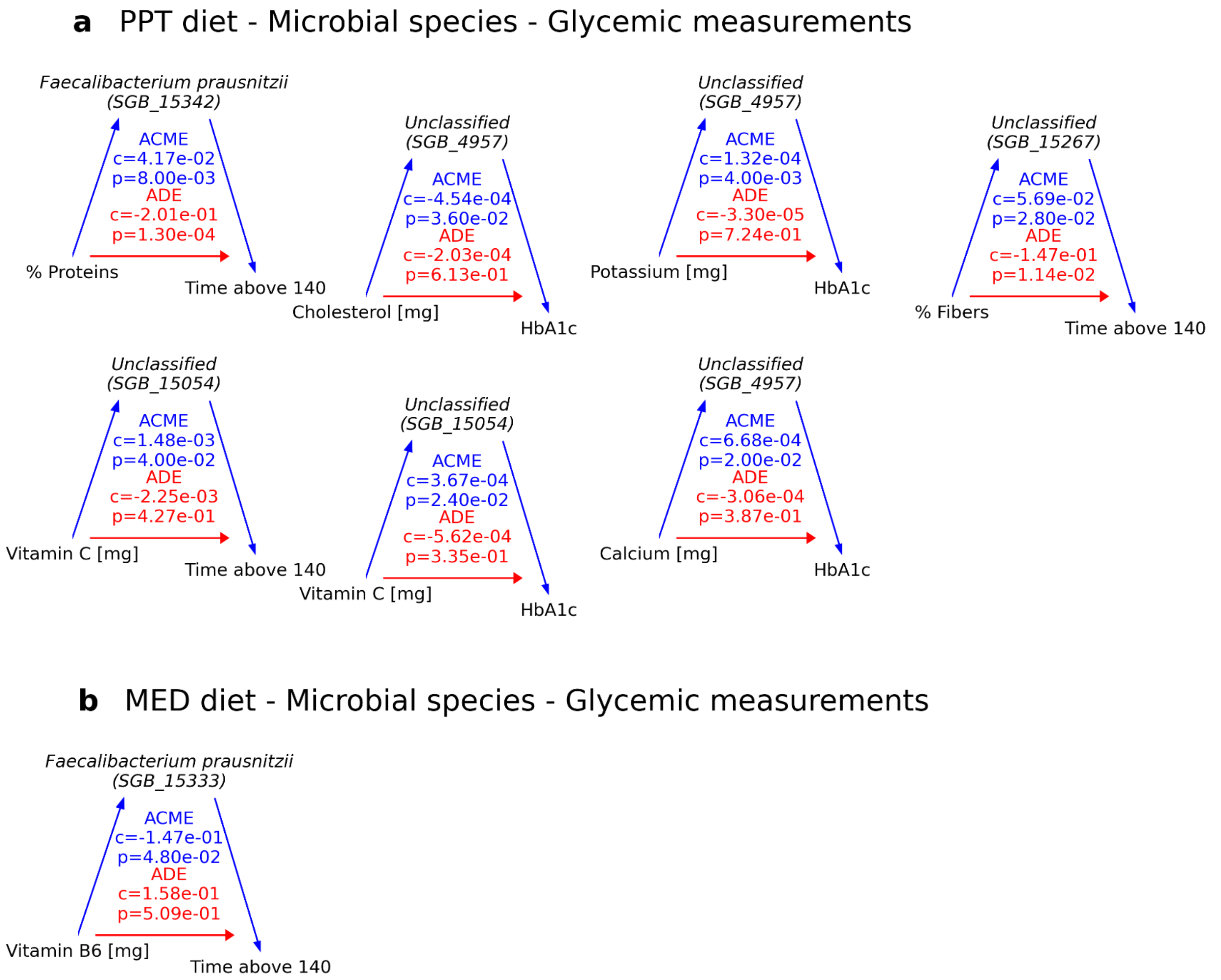

a PPT diet - Microbial species - Glycemic measurements
Faecalibacterium prausnitzii (SGB_15342)
ACME c=4.17e-02 p=8.00e-03
ADE c=-2.01e-01 p=1.30e-04
% Proteins
Time above 140
Unclassified (SGB_4957)
ACME c=-4.54e-04 p=3.60e-02
ADE c=-2.03e-04 p=6.13e-01
Cholesterol [mg]
HbA1c
Unclassified (SGB_4957)
ACME c=1.32e-04 p=4.00e-03
ADE c=-3.30e-05 p=7.24e-01
Potassium [mg]
HbA1c
Unclassified (SGB_15267)
ACME c=5.69e-02 p=2.80e-02
ADE c=-1.47e-01 p=1.14e-02
% Fibers
Time above 140
Unclassified (SGB_15054)
ACME c=1.48e-03 p=4.00e-02
ADE c=-2.25e-03 p=4.27e-01
Vitamin C [mg]
Time above 140
Unclassified (SGB_15054)
ACME c=3.67e-04 p=2.40e-02
ADE c=-5.62e-04 p=3.35e-01
Vitamin C [mg]
HbA1c
Unclassified (SGB_4957)
ACME c=6.68e-04 p=2.00e-02
ADE c=-3.06e-04 p=3.87e-01
Calcium [mg]
HbA1c
b MED diet - Microbial species - Glycemic measurements
Faecalibacterium prausnitzii (SGB_15333)
ACME c=-1.47e-01 p=4.80e-02
ADE c=1.58e-01 p=5.09e-01
Vitamin B6 [mg]
Time above 140

c PPT diet - Microbial species - Metabolites and Cytokines

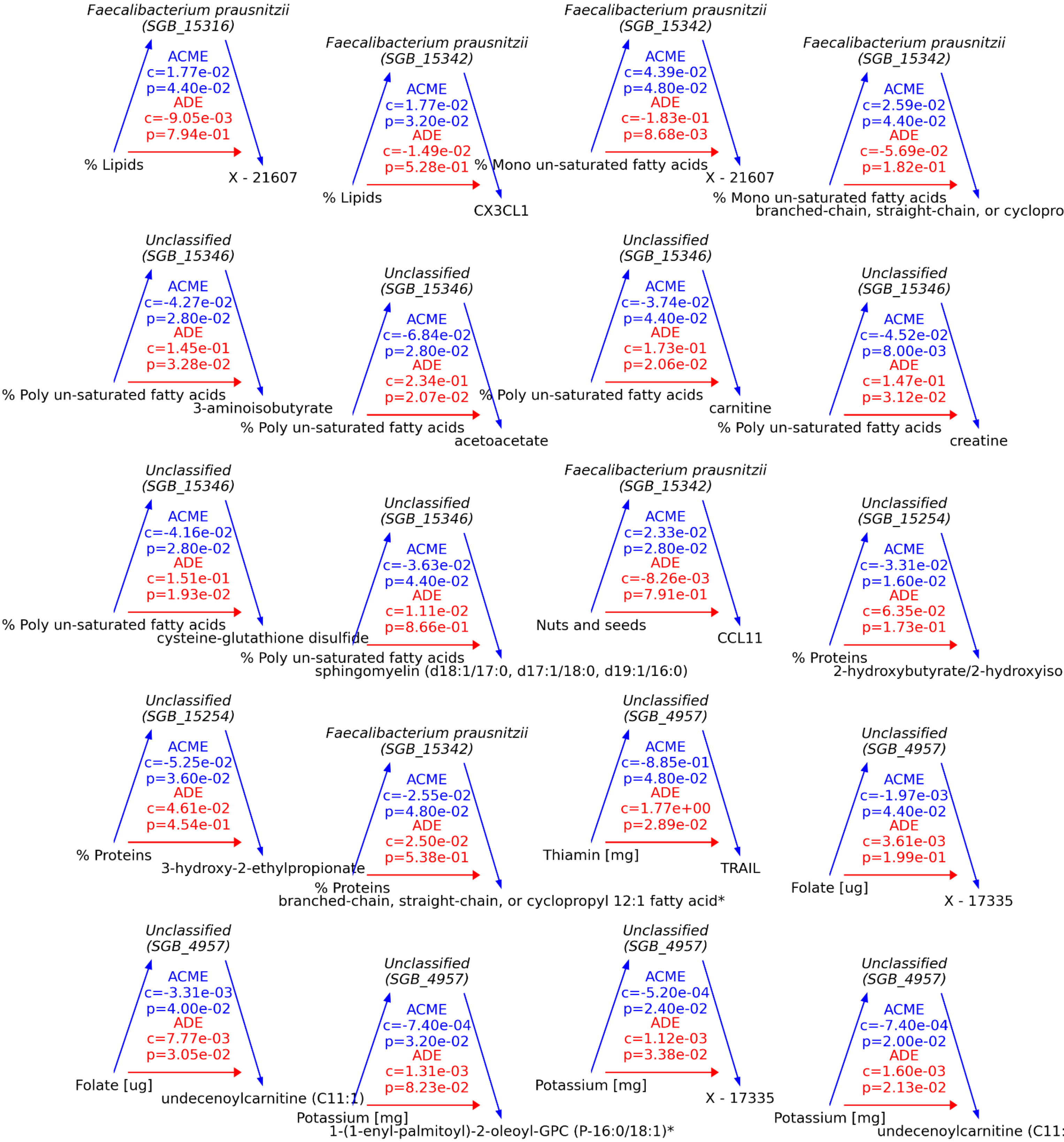

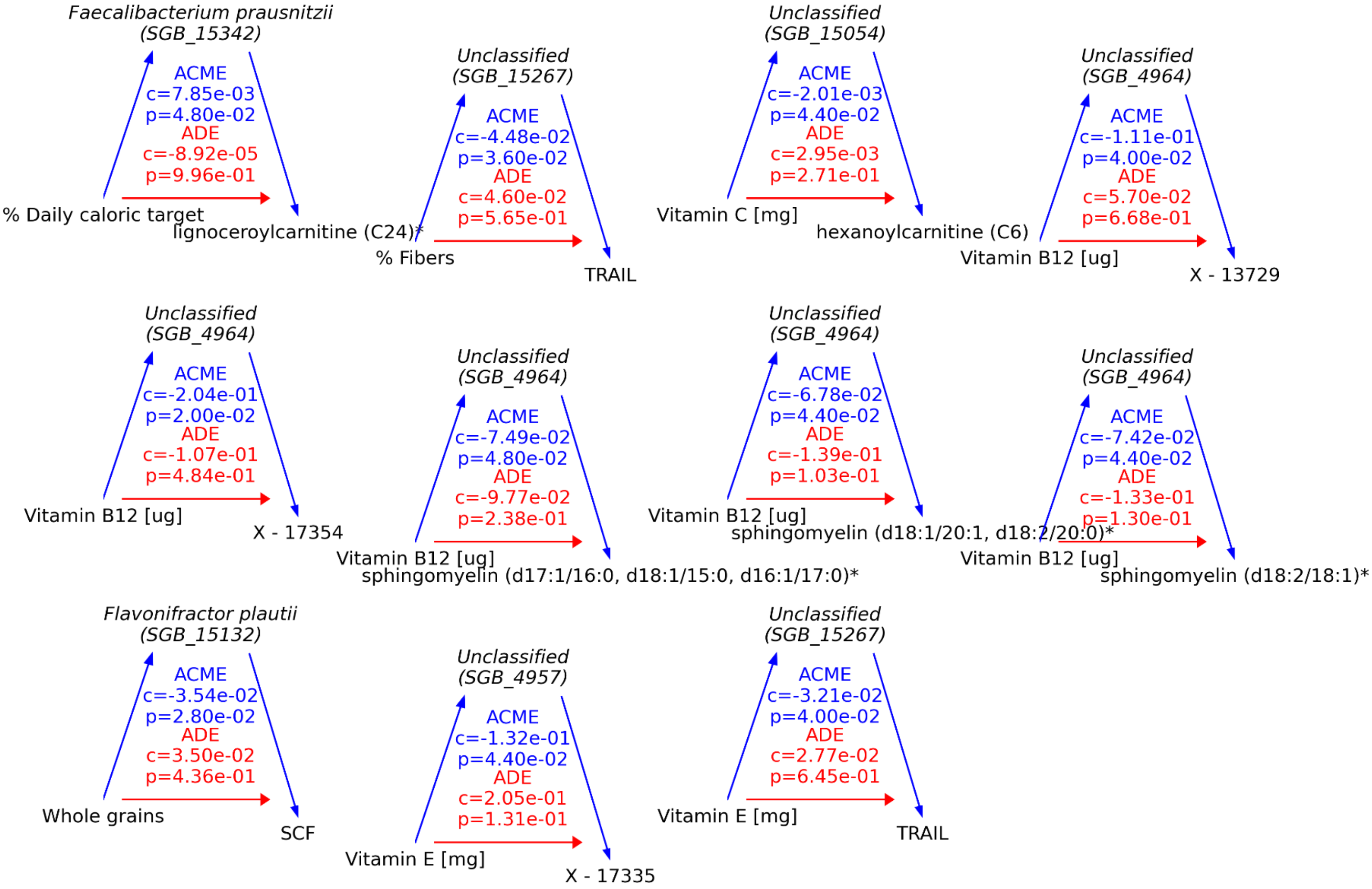

d MED diet - Microbial species - Metabolites and Cytokines

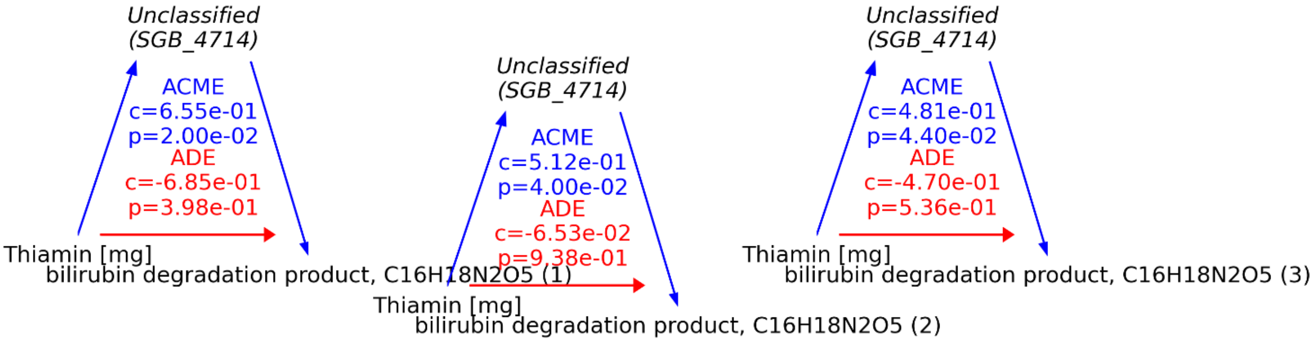

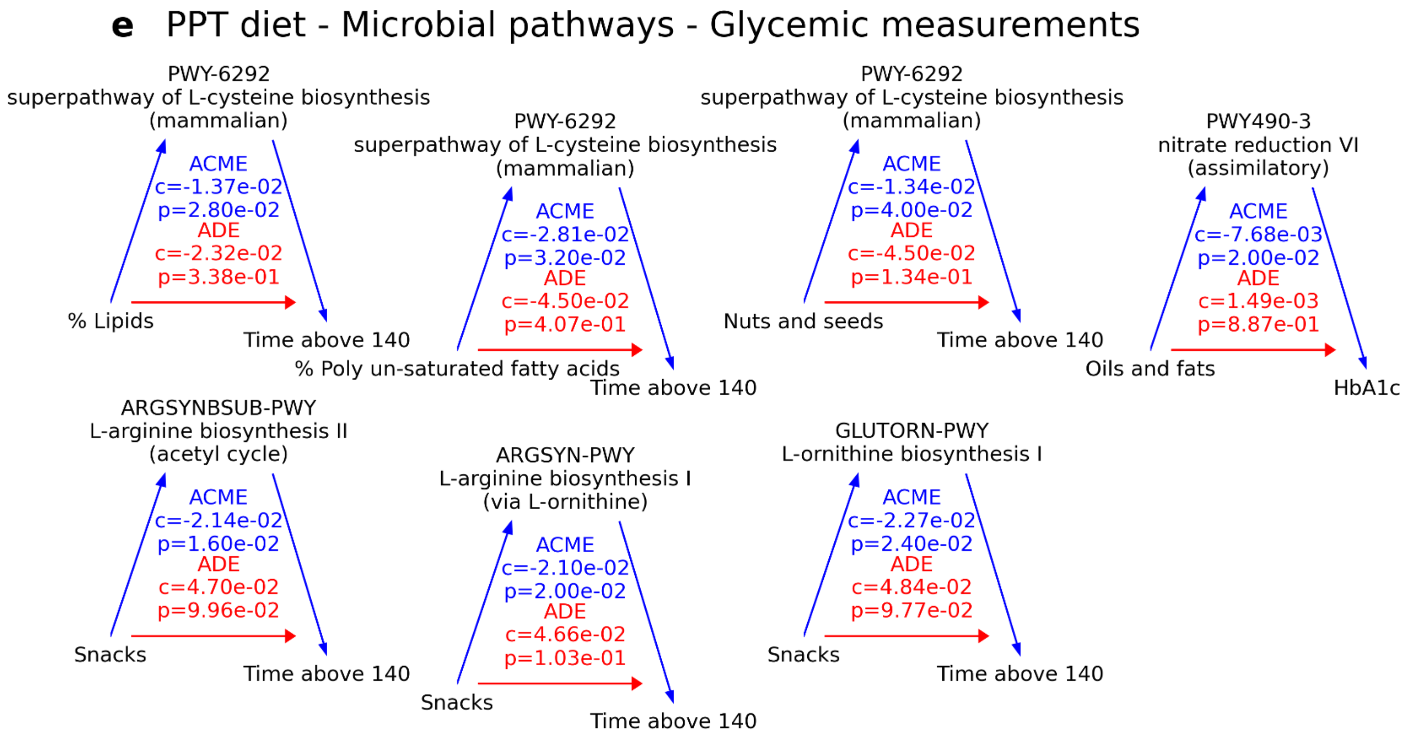

e PPT diet - Microbial pathways - Glycemic measurements
PWY-6292
superpathway of L-cysteine biosynthesis
(mammalian)
ACME
c=-1.37e-02
p=2.80e-02
ADE
c=-2.32e-02
p=3.38e-01
% Lipids
Time above 140
PWY-6292
superpathway of L-cysteine biosynthesis
(mammalian)
ACME
c=-2.81e-02
p=3.20e-02
ADE
c=-4.50e-02
p=4.07e-01
% Poly un-saturated fatty acids
Time above 140
PWY-6292
superpathway of L-cysteine biosynthesis
(mammalian)
ACME
c=-1.34e-02
p=4.00e-02
ADE
c=-4.50e-02
p=1.34e-01
Nuts and seeds
Time above 140
PWY490-3
nitrate reduction VI
(assimilatory)
ACME
c=-7.68e-03
p=2.00e-02
ADE
c=1.49e-03
p=8.87e-01
Oils and fats
HbA1c
ARGSYNBSUB-PWY
L-arginine biosynthesis II
(acetyl cycle)
ACME
c=-2.14e-02
p=1.60e-02
ADE
c=4.70e-02
p=9.96e-02
Snacks
Time above 140
ARGSYN-PWY
L-arginine biosynthesis I
(via L-ornithine)
ACME
c=-2.10e-02
p=2.00e-02
ADE
c=4.66e-02
p=1.03e-01
Snacks
Time above 140
GLUTORN-PWY
L-ornithine biosynthesis I
ACME
c=-2.27e-02
p=2.40e-02
ADE
c=4.84e-02
p=9.77e-02
Snacks
Time above 140

f PPT diet - Microbial pathways - Metabolites and Cytokines

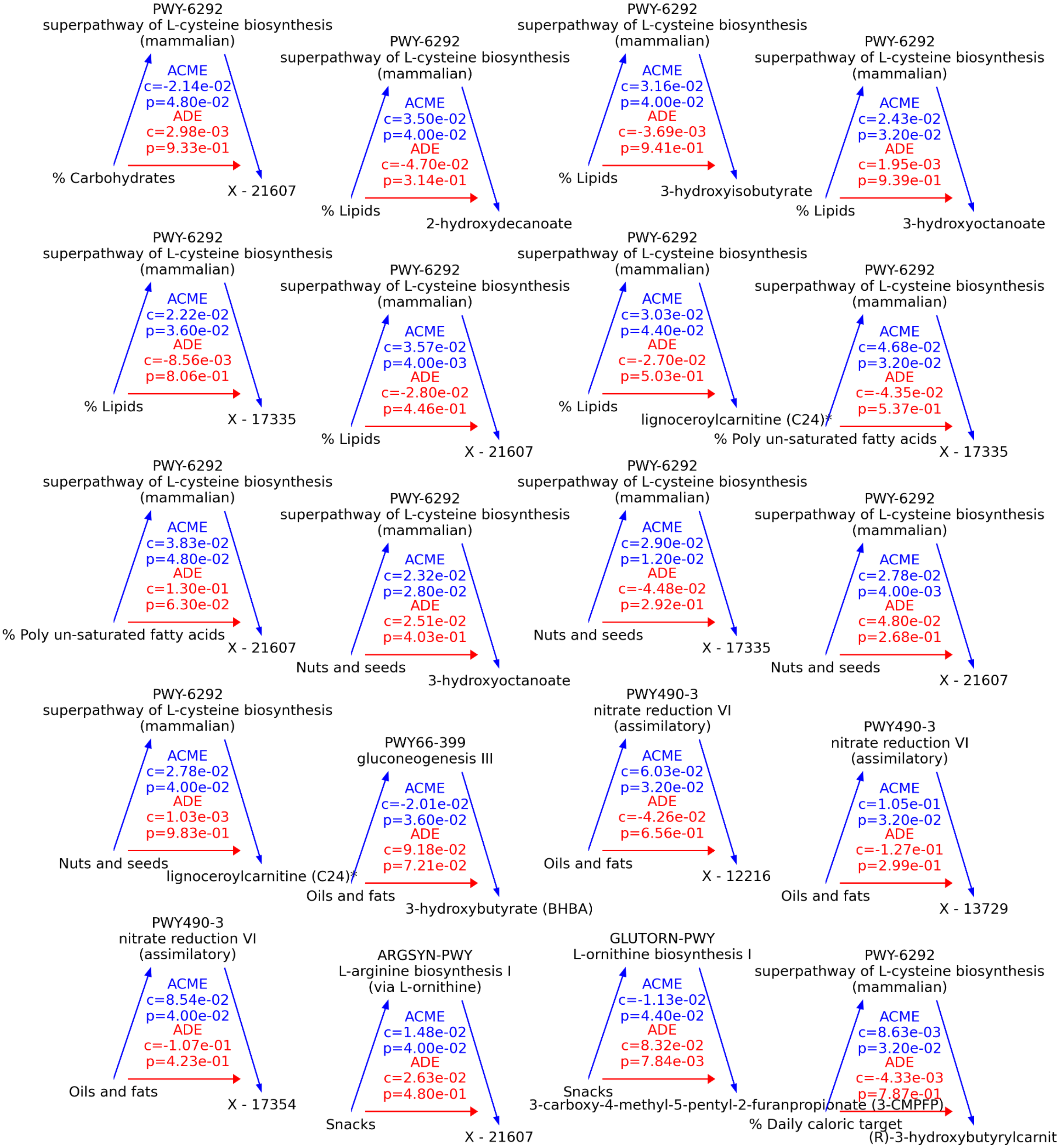

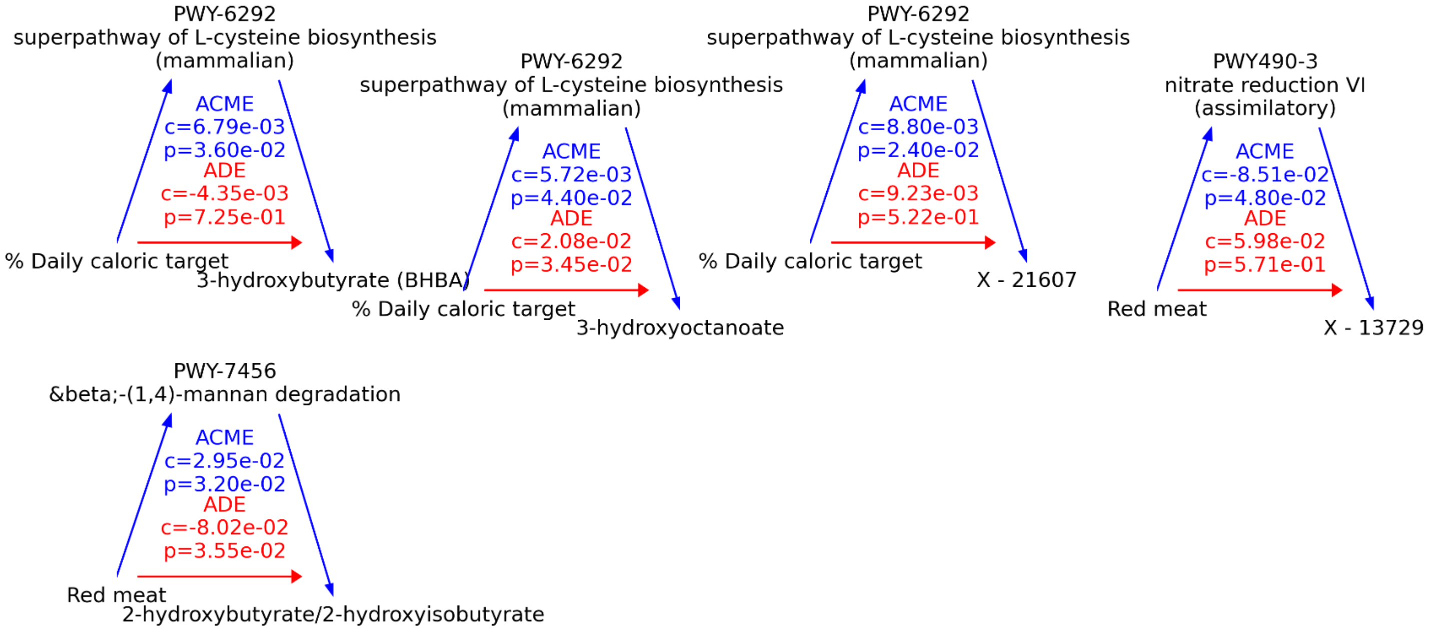
PWY-6292
superpathway of L-cysteine biosynthesis
(mammalian)
ACME
c=6.79e-03
p=3.60e-02
ADE
c=-4.35e-03
p=7.25e-01
% Daily caloric target
3-hydroxybutyrate (BHBA)
PWY-6292
superpathway of L-cysteine biosynthesis
(mammalian)
ACME
c=5.72e-03
p=4.40e-02
ADE
c=2.08e-02
p=3.45e-02
% Daily caloric target
3-hydroxyoctanoate
PWY-6292
superpathway of L-cysteine biosynthesis
(mammalian)
ACME
c=8.80e-03
p=2.40e-02
ADE
c=9.23e-03
p=5.22e-01
% Daily caloric target
X - 21607
PWY490-3
nitrate reduction VI
(assimilatory)
ACME
c=-8.51e-02
p=4.80e-02
ADE
c=5.98e-02
p=5.71e-01
Red meat
X - 13729
PWY-7456
β-(1,4)-mannan degradation
ACME
c=2.95e-02
p=3.20e-02
ADE
c=-8.02e-02
p=3.55e-02
Red meat
2-hydroxybutyrate/2-hydroxyisobutyrate

g MED diet - Microbial pathways - Metabolites and Cytokines

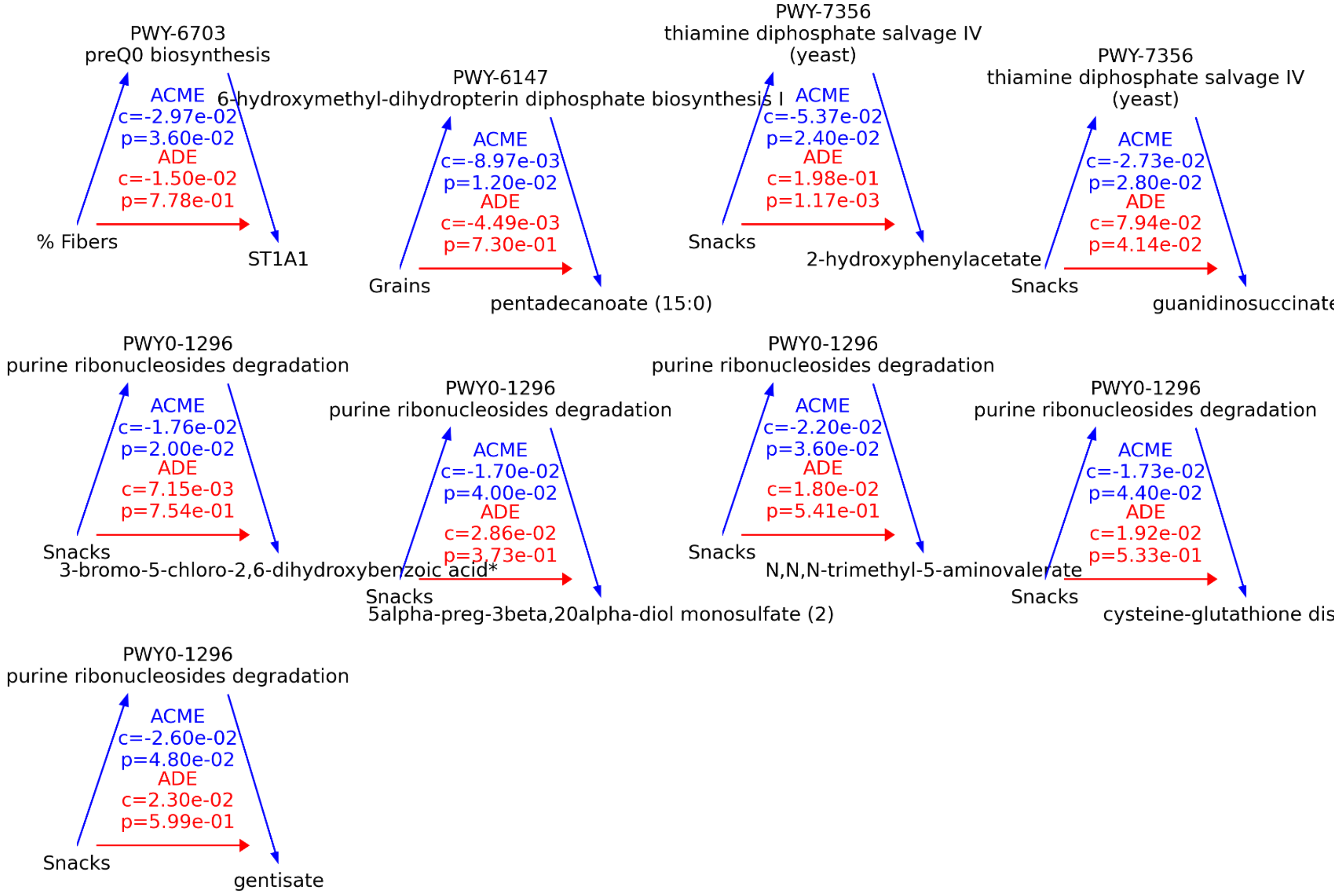

h Diet - Oral strains - Glycemic measurements

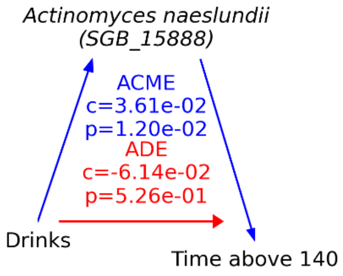

i Diet - Gut strains - Metabolites and Cytokines

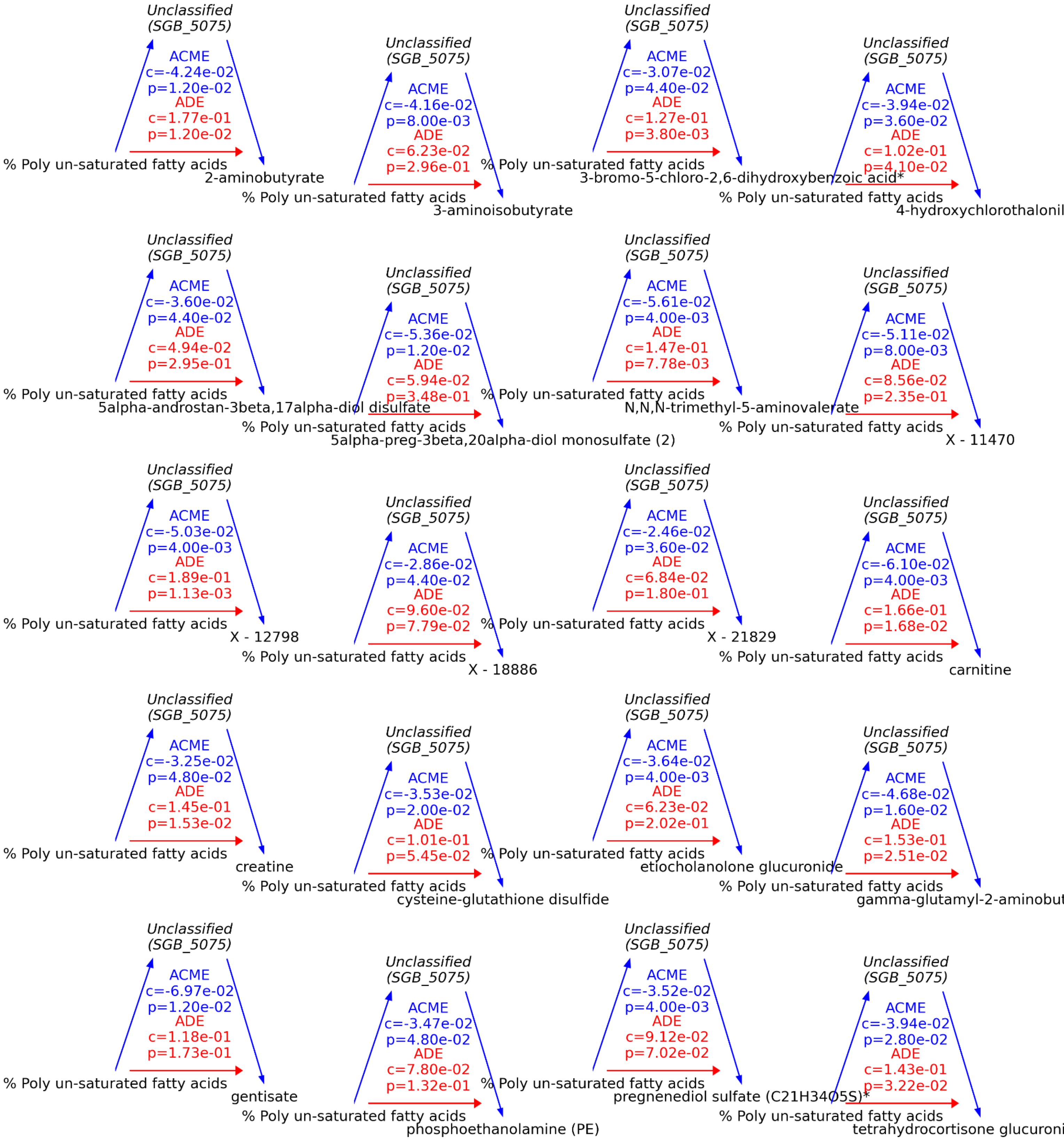

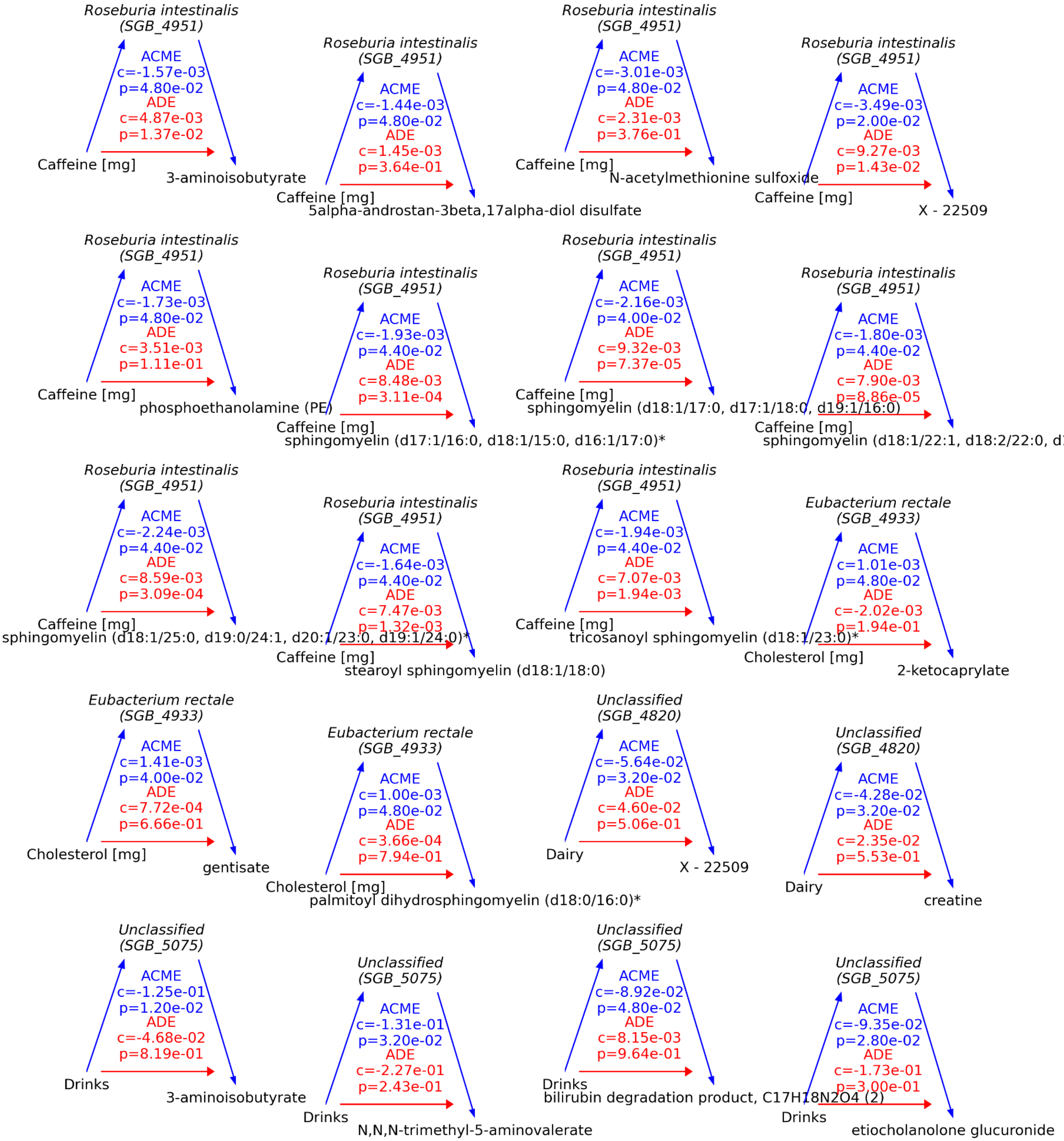

Roseburia intestinalis (SGB_4951)
ACME c=-1.57e-03 p=4.80e-02
ADE c=4.87e-03 p=1.37e-02
Caffeine [mg]
3-aminoisobutyrate
Roseburia intestinalis (SGB_4951)
ACME c=-1.44e-03 p=4.80e-02
ADE c=1.45e-03 p=3.64e-01
Caffeine [mg]
5alpha-androstan-3beta,17alpha-diol disulfate
Roseburia intestinalis (SGB_4951)
ACME c=-3.01e-03 p=4.80e-02
ADE c=2.31e-03 p=3.76e-01
Caffeine [mg]
N-acetylmethionine sulfoxide
Roseburia intestinalis (SGB_4951)
ACME c=-3.49e-03 p=2.00e-02
ADE c=9.27e-03 p=1.43e-02
Caffeine [mg]
X - 22509
Roseburia intestinalis (SGB_4951)
ACME c=-1.73e-03 p=4.80e-02
ADE c=3.51e-03 p=1.11e-01
Caffeine [mg]
phosphoethanolamine (PE)
Roseburia intestinalis (SGB_4951)
ACME c=-1.93e-03 p=4.40e-02
ADE c=8.48e-03 p=3.11e-04
Caffeine [mg]
sphingomyelin (d17:1/16:0, d18:1/15:0, d16:1/17:0)*
Roseburia intestinalis (SGB_4951)
ACME c=-2.16e-03 p=4.00e-02
ADE c=9.32e-03 p=7.37e-05
Caffeine [mg]
sphingomyelin (d18:1/17:0, d17:1/18:0, d19:1/16:0)
Roseburia intestinalis (SGB_4951)
ACME c=-1.80e-03 p=4.40e-02
ADE c=7.90e-03 p=8.86e-05
Caffeine [mg]
sphingomyelin (d18:1/22:1, d18:2/22:0, d1
Roseburia intestinalis (SGB_4951)
ACME c=-2.24e-03 p=4.40e-02
ADE c=8.59e-03 p=3.09e-04
Caffeine [mg]
sphingomyelin (d18:1/25:0, d19:0/24:1, d20:1/23:0, d19:1/24:0)*
Roseburia intestinalis (SGB_4951)
ACME c=-1.64e-03 p=4.40e-02
ADE c=7.47e-03 p=1.32e-03
Caffeine [mg]
stearoyl sphingomyelin (d18:1/18:0)
Roseburia intestinalis (SGB_4951)
ACME c=-1.94e-03 p=4.40e-02
ADE c=7.07e-03 p=1.94e-03
Caffeine [mg]
tricosanoyl sphingomyelin (d18:1/23:0)*
Eubacterium rectale (SGB_4933)
ACME c=1.01e-03 p=4.80e-02
ADE c=-2.02e-03 p=1.94e-01
Cholesterol [mg]
2-ketocaprylate
Eubacterium rectale (SGB_4933)
ACME c=1.41e-03 p=4.00e-02
ADE c=7.72e-04 p=6.66e-01
Cholesterol [mg]
gentisate
Eubacterium rectale (SGB_4933)
ACME c=1.00e-03 p=4.80e-02
ADE c=3.66e-04 p=7.94e-01
Cholesterol [mg]
palmitoyl dihydrosphingomyelin (d18:0/16:0)*
Unclassified (SGB_4820)
ACME c=-5.64e-02 p=3.20e-02
ADE c=4.60e-02 p=5.06e-01
Dairy
X - 22509
Unclassified (SGB_4820)
ACME c=-4.28e-02 p=3.20e-02
ADE c=2.35e-02 p=5.53e-01
Dairy
creatine
Unclassified (SGB_5075)
ACME c=-1.25e-01 p=1.20e-02
ADE c=-4.68e-02 p=8.19e-01
Drinks
3-aminoisobutyrate
Unclassified (SGB_5075)
ACME c=-1.31e-01 p=3.20e-02
ADE c=-2.27e-01 p=2.43e-01
Drinks
N,N,N-trimethyl-5-aminovalerate
Unclassified (SGB_5075)
ACME c=-8.92e-02 p=4.80e-02
ADE c=8.15e-03 p=9.64e-01
Drinks
bilirubin degradation product, C17H18N2O4 (2)
Unclassified (SGB_5075)
ACME c=-9.35e-02 p=2.80e-02
ADE c=-1.73e-01 p=3.00e-01
Drinks
etiocholanolone glucuronide

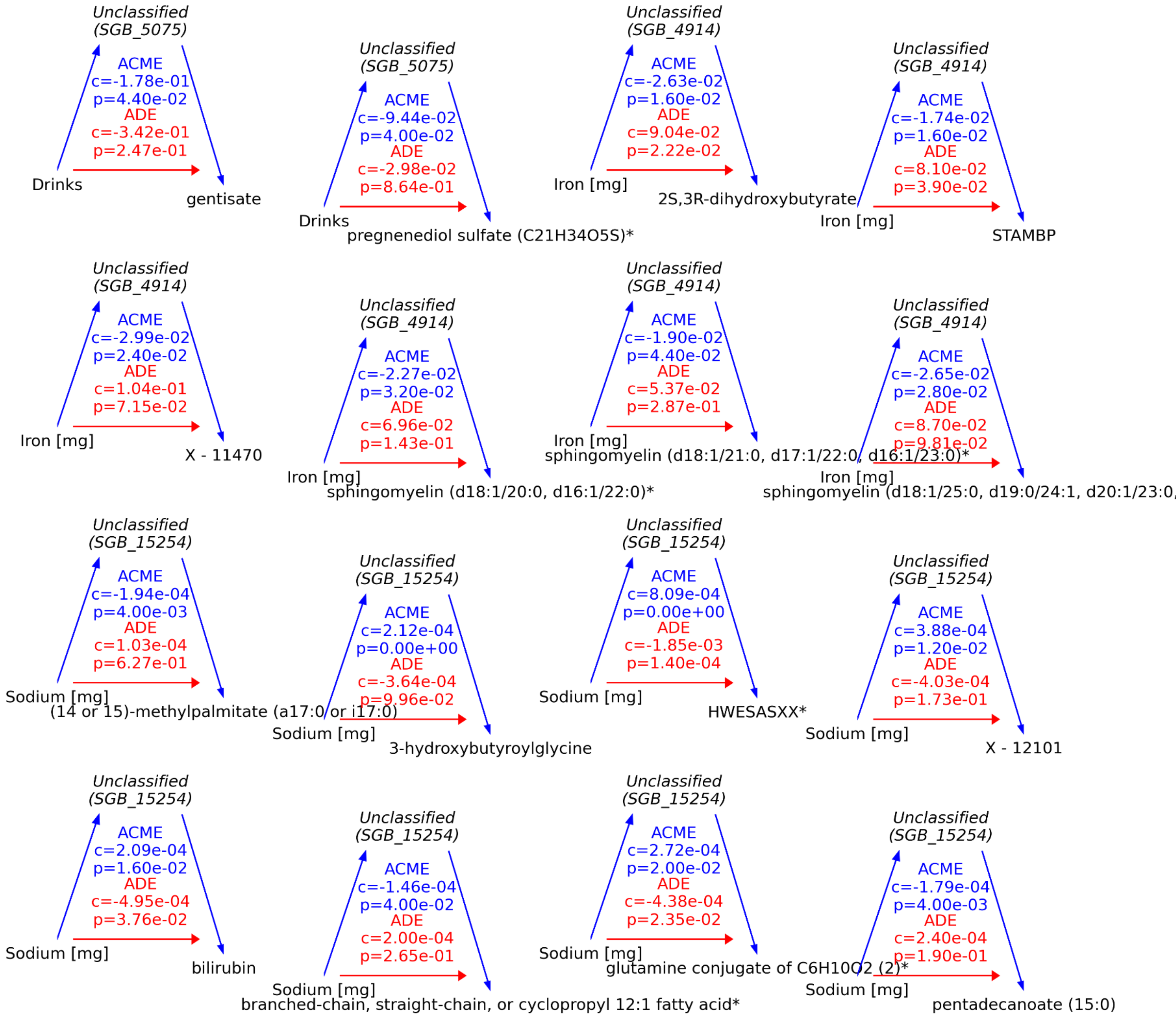
Unclassified (SGB_5075)
ACME c=-1.78e-01 p=4.40e-02
ADE c=-3.42e-01 p=2.47e-01
Drinks
gentisate
Unclassified (SGB_5075)
ACME c=-9.44e-02 p=4.00e-02
ADE c=-2.98e-02 p=8.64e-01
Drinks
pregnenediol sulfate (C21H34O5S)*
Unclassified (SGB_4914)
ACME c=-2.63e-02 p=1.60e-02
ADE c=9.04e-02 p=2.22e-02
Iron [mg]
2S,3R-dihydroxybutyrate
Unclassified (SGB_4914)
ACME c=-1.74e-02 p=1.60e-02
ADE c=8.10e-02 p=3.90e-02
Iron [mg]
STAMBP
Unclassified (SGB_4914)
ACME c=-2.99e-02 p=2.40e-02
ADE c=1.04e-01 p=7.15e-02
Iron [mg]
X - 11470
Unclassified (SGB_4914)
ACME c=-2.27e-02 p=3.20e-02
ADE c=6.96e-02 p=1.43e-01
Iron [mg]
sphingomyelin (d18:1/20:0, d16:1/22:0)*
Unclassified (SGB_4914)
ACME c=-1.90e-02 p=4.40e-02
ADE c=5.37e-02 p=2.87e-01
Iron [mg]
sphingomyelin (d18:1/21:0, d17:1/22:0, d16:1/23:0)*
Unclassified (SGB_4914)
ACME c=-2.65e-02 p=2.80e-02
ADE c=8.70e-02 p=9.81e-02
Iron [mg]
sphingomyelin (d18:1/25:0, d19:0/24:1, d20:1/23:0,
Unclassified (SGB_15254)
ACME c=-1.94e-04 p=4.00e-03
ADE c=1.03e-04 p=6.27e-01
Sodium [mg]
(14 or 15)-methylpalmitate (a17:0 or i17:0)
Unclassified (SGB_15254)
ACME c=2.12e-04 p=0.00e+00
ADE c=-3.64e-04 p=9.96e-02
Sodium [mg]
3-hydroxybutyroylglycine
Unclassified (SGB_15254)
ACME c=8.09e-04 p=0.00e+00
ADE c=-1.85e-03 p=1.40e-04
Sodium [mg]
HWESASXX*
Unclassified (SGB_15254)
ACME c=3.88e-04 p=1.20e-02
ADE c=-4.03e-04 p=1.73e-01
Sodium [mg]
X - 12101
Unclassified (SGB_15254)
ACME c=2.09e-04 p=1.60e-02
ADE c=-4.95e-04 p=3.76e-02
Sodium [mg]
bilirubin
Unclassified (SGB_15254)
ACME c=-1.46e-04 p=4.00e-02
ADE c=2.00e-04 p=2.65e-01
Sodium [mg]
branched-chain, straight-chain, or cyclopropyl 12:1 fatty acid*
Unclassified (SGB_15254)
ACME c=2.72e-04 p=2.00e-02
ADE c=-4.38e-04 p=2.35e-02
Sodium [mg]
glutamine conjugate of C6H10O2 (2)*
Unclassified (SGB_15254)
ACME c=-1.79e-04 p=4.00e-03
ADE c=2.40e-04 p=1.90e-01
Sodium [mg]
pentadecanoate (15:0)

j Diet - Oral strains - Metabolites and Cytokines

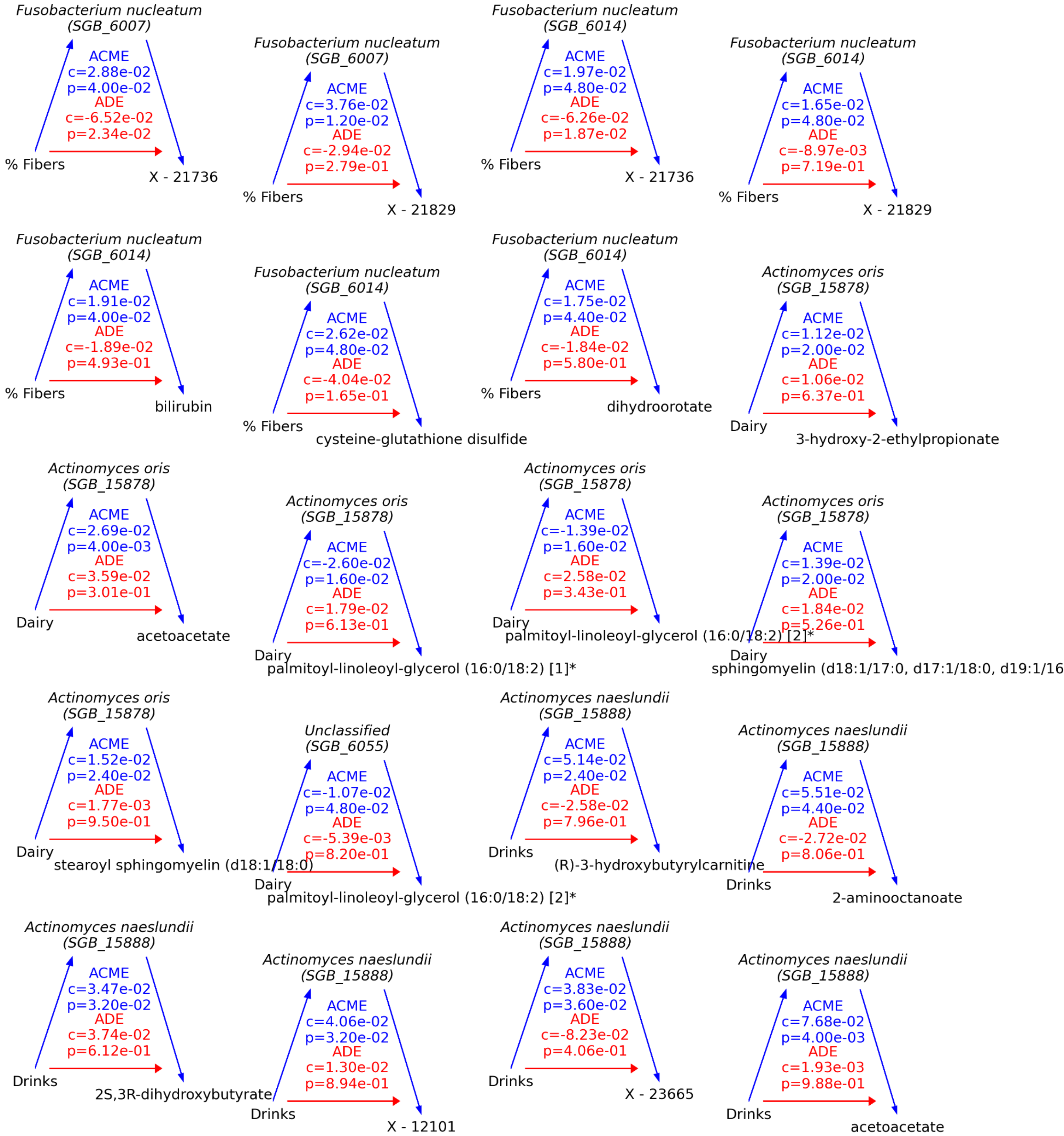

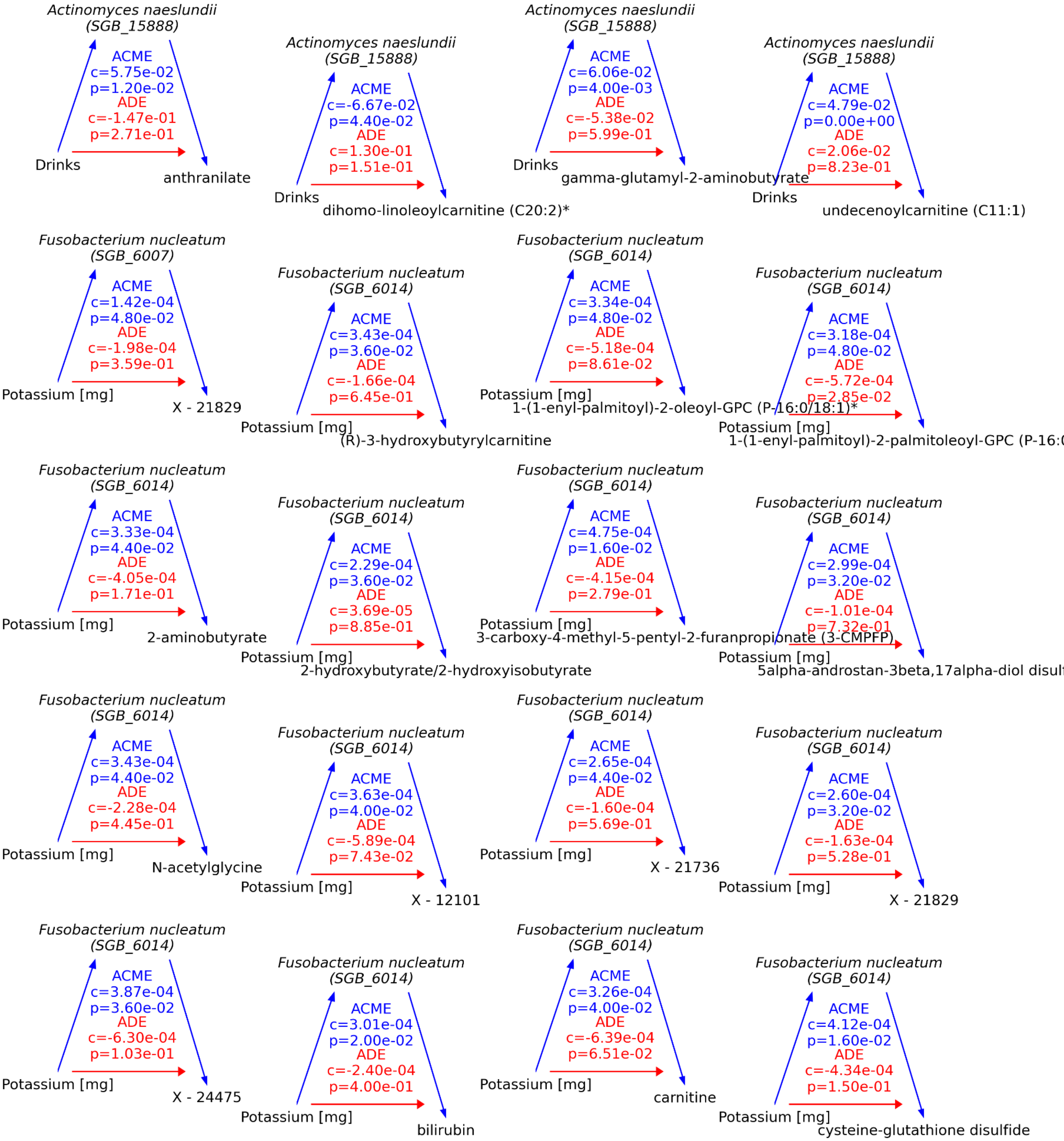
Actinomyces naeslundii (SGB_15888)
ACME c=5.75e-02 p=1.20e-02
ADE c=-1.47e-01 p=2.71e-01
Drinks
anthranilate
Actinomyces naeslundii (SGB_15888)
ACME c=-6.67e-02 p=4.40e-02
ADE c=1.30e-01 p=1.51e-01
Drinks
dihomo-linoleoylcarnitine (C20:2)*
Actinomyces naeslundii (SGB_15888)
ACME c=6.06e-02 p=4.00e-03
ADE c=-5.38e-02 p=5.99e-01
Drinks
gamma-glutamyl-2-aminobutyrate
Actinomyces naeslundii (SGB_15888)
ACME c=4.79e-02 p=0.00e+00
ADE c=2.06e-02 p=8.23e-01
Drinks
undecenoylcarnitine (C11:1)
Fusobacterium nucleatum (SGB_6007)
ACME c=1.42e-04 p=4.80e-02
ADE c=-1.98e-04 p=3.59e-01
Potassium [mg]
X - 21829
Fusobacterium nucleatum (SGB_6014)
ACME c=3.43e-04 p=3.60e-02
ADE c=-1.66e-04 p=6.45e-01
Potassium [mg]
(R)-3-hydroxybutyrylcarnitine
Fusobacterium nucleatum (SGB_6014)
ACME c=3.34e-04 p=4.80e-02
ADE c=-5.18e-04 p=8.61e-02
Potassium [mg]
1-(1-enyl-palmitoyl)-2-oleoyl-GPC (P-16:0/18:1)*
Fusobacterium nucleatum (SGB_6014)
ACME c=3.18e-04 p=4.80e-02
ADE c=-5.72e-04 p=2.85e-02
Potassium [mg]
1-(1-enyl-palmitoyl)-2-palmitoleoyl-GPC (P-16:
Fusobacterium nucleatum (SGB_6014)
ACME c=3.33e-04 p=4.40e-02
ADE c=-4.05e-04 p=1.71e-01
Potassium [mg]
2-aminobutyrate
Fusobacterium nucleatum (SGB_6014)
ACME c=2.29e-04 p=3.60e-02
ADE c=3.69e-05 p=8.85e-01
Potassium [mg]
2-hydroxybutyrate/2-hydroxyisobutyrate
Fusobacterium nucleatum (SGB_6014)
ACME c=4.75e-04 p=1.60e-02
ADE c=-4.15e-04 p=2.79e-01
Potassium [mg]
3-carboxy-4-methyl-5-pentyl-2-furanpropionate (3-CMPFP)
Fusobacterium nucleatum (SGB_6014)
ACME c=2.99e-04 p=3.20e-02
ADE c=-1.01e-04 p=7.32e-01
Potassium [mg]
5alpha-androstan-3beta,17alpha-diol disul
Fusobacterium nucleatum (SGB_6014)
ACME c=3.43e-04 p=4.40e-02
ADE c=-2.28e-04 p=4.45e-01
Potassium [mg]
N-acetylglycine
Fusobacterium nucleatum (SGB_6014)
ACME c=3.63e-04 p=4.00e-02
ADE c=-5.89e-04 p=7.43e-02
Potassium [mg]
X - 12101
Fusobacterium nucleatum (SGB_6014)
ACME c=2.65e-04 p=4.40e-02
ADE c=-1.60e-04 p=5.69e-01
Potassium [mg]
X - 21736
Fusobacterium nucleatum (SGB_6014)
ACME c=2.60e-04 p=3.20e-02
ADE c=-1.63e-04 p=5.28e-01
Potassium [mg]
X - 21829
Fusobacterium nucleatum (SGB_6014)
ACME c=3.87e-04 p=3.60e-02
ADE c=-6.30e-04 p=1.03e-01
Potassium [mg]
X - 24475
Fusobacterium nucleatum (SGB_6014)
ACME c=3.01e-04 p=2.00e-02
ADE c=-2.40e-04 p=4.00e-01
Potassium [mg]
bilirubin
Fusobacterium nucleatum (SGB_6014)
ACME c=3.26e-04 p=4.00e-02
ADE c=-6.39e-04 p=6.51e-02
Potassium [mg]
carnitine
Fusobacterium nucleatum (SGB_6014)
ACME c=4.12e-04 p=1.60e-02
ADE c=-4.34e-04 p=1.50e-01
Potassium [mg]
cysteine-glutathione disulfide

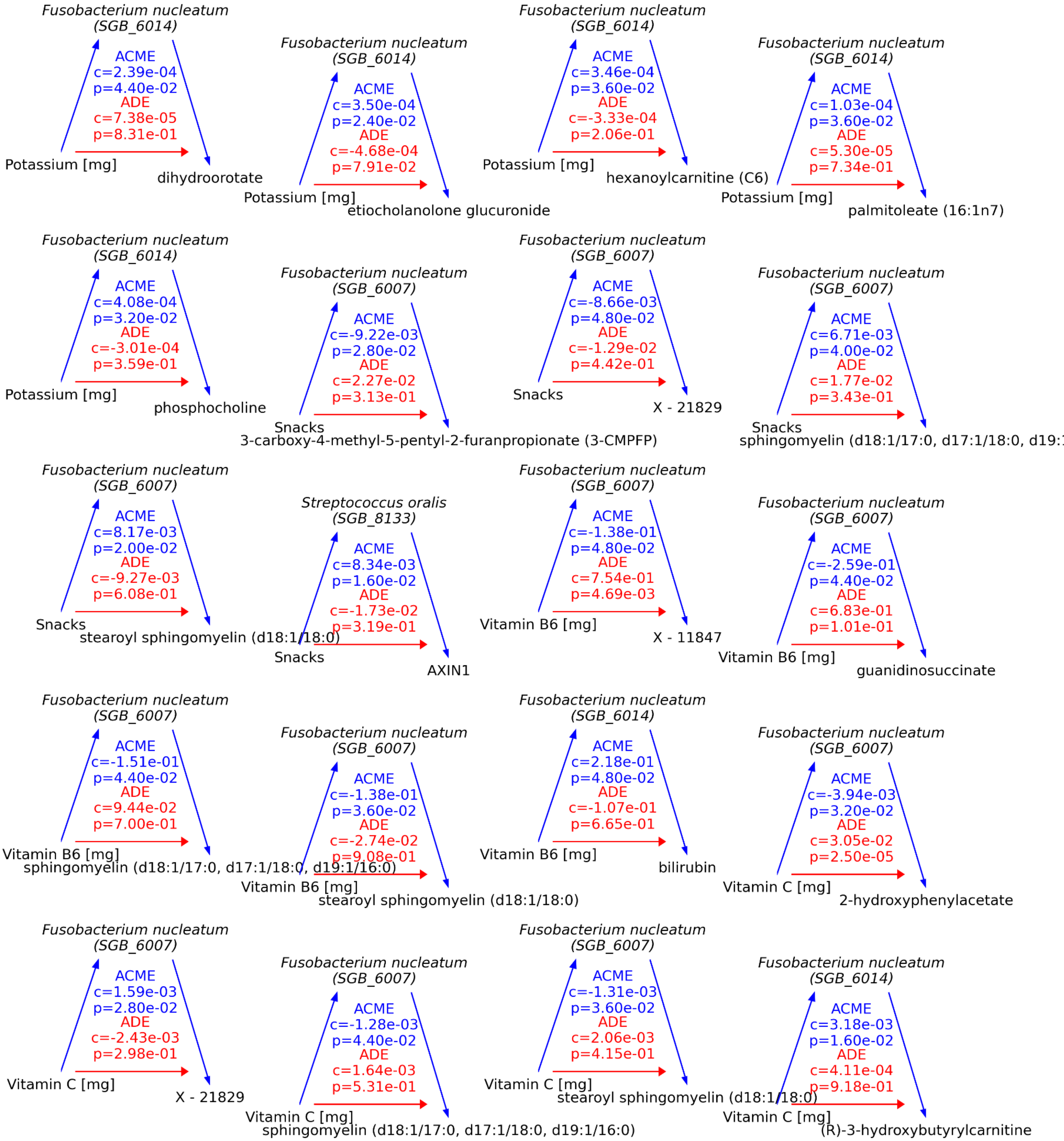
Fusobacterium nucleatum (SGB_6014)
ACME c=2.39e-04 p=4.40e-02
ADE c=7.38e-05 p=8.31e-01
Potassium [mg]
dihydroorotate
Fusobacterium nucleatum (SGB_6014)
ACME c=3.50e-04 p=2.40e-02
ADE c=-4.68e-04 p=7.91e-02
Potassium [mg]
etiocholanolone glucuronide
Fusobacterium nucleatum (SGB_6014)
ACME c=3.46e-04 p=3.60e-02
ADE c=-3.33e-04 p=2.06e-01
Potassium [mg]
hexanoylcarnitine (C6)
Fusobacterium nucleatum (SGB_6014)
ACME c=1.03e-04 p=3.60e-02
ADE c=5.30e-05 p=7.34e-01
Potassium [mg]
palmitoleate (16:1n7)
Fusobacterium nucleatum (SGB_6014)
ACME c=4.08e-04 p=3.20e-02
ADE c=-3.01e-04 p=3.59e-01
Potassium [mg]
phosphocholine
Fusobacterium nucleatum (SGB_6007)
ACME c=-9.22e-03 p=2.80e-02
ADE c=2.27e-02 p=3.13e-01
Snacks
3-carboxy-4-methyl-5-pentyl-2-furanpropionate (3-CMPFP)
Fusobacterium nucleatum (SGB_6007)
ACME c=-8.66e-03 p=4.80e-02
ADE c=-1.29e-02 p=4.42e-01
Snacks
X - 21829
Fusobacterium nucleatum (SGB_6007)
ACME c=6.71e-03 p=4.00e-02
ADE c=1.77e-02 p=3.43e-01
Snacks
sphingomyelin (d18:1/17:0, d17:1/18:0, d19:
Fusobacterium nucleatum (SGB_6007)
ACME c=8.17e-03 p=2.00e-02
ADE c=-9.27e-03 p=6.08e-01
Snacks
stearoyl sphingomyelin (d18:1/18:0)
Streptococcus oralis (SGB_8133)
ACME c=8.34e-03 p=1.60e-02
ADE c=-1.73e-02 p=3.19e-01
Snacks
AXIN1
Fusobacterium nucleatum (SGB_6007)
ACME c=-1.38e-01 p=4.80e-02
ADE c=7.54e-01 p=4.69e-03
Vitamin B6 [mg]
X - 11847
Fusobacterium nucleatum (SGB_6007)
ACME c=-2.59e-01 p=4.40e-02
ADE c=6.83e-01 p=1.01e-01
Vitamin B6 [mg]
guanidinosuccinate
Fusobacterium nucleatum (SGB_6007)
ACME c=-1.51e-01 p=4.40e-02
ADE c=9.44e-02 p=7.00e-01
Vitamin B6 [mg]
sphingomyelin (d18:1/17:0, d17:1/18:0, d19:1/16:0)
Fusobacterium nucleatum (SGB_6007)
ACME c=-1.38e-01 p=3.60e-02
ADE c=-2.74e-02 p=9.08e-01
Vitamin B6 [mg]
stearoyl sphingomyelin (d18:1/18:0)
Fusobacterium nucleatum (SGB_6014)
ACME c=2.18e-01 p=4.80e-02
ADE c=-1.07e-01 p=6.65e-01
Vitamin B6 [mg]
bilirubin
Fusobacterium nucleatum (SGB_6007)
ACME c=-3.94e-03 p=3.20e-02
ADE c=3.05e-02 p=2.50e-05
Vitamin C [mg]
2-hydroxyphenylacetate
Fusobacterium nucleatum (SGB_6007)
ACME c=1.59e-03 p=2.80e-02
ADE c=-2.43e-03 p=2.98e-01
Vitamin C [mg]
X - 21829
Fusobacterium nucleatum (SGB_6007)
ACME c=-1.28e-03 p=4.40e-02
ADE c=1.64e-03 p=5.31e-01
Vitamin C [mg]
sphingomyelin (d18:1/17:0, d17:1/18:0, d19:1/16:0)
Fusobacterium nucleatum (SGB_6007)
ACME c=-1.31e-03 p=3.60e-02
ADE c=2.06e-03 p=4.15e-01
Vitamin C [mg]
stearoyl sphingomyelin (d18:1/18:0)
Fusobacterium nucleatum (SGB_6014)
ACME c=3.18e-03 p=1.60e-02
ADE c=4.11e-04 p=9.18e-01
Vitamin C [mg]
(R)-3-hydroxybutyrylcarnitine

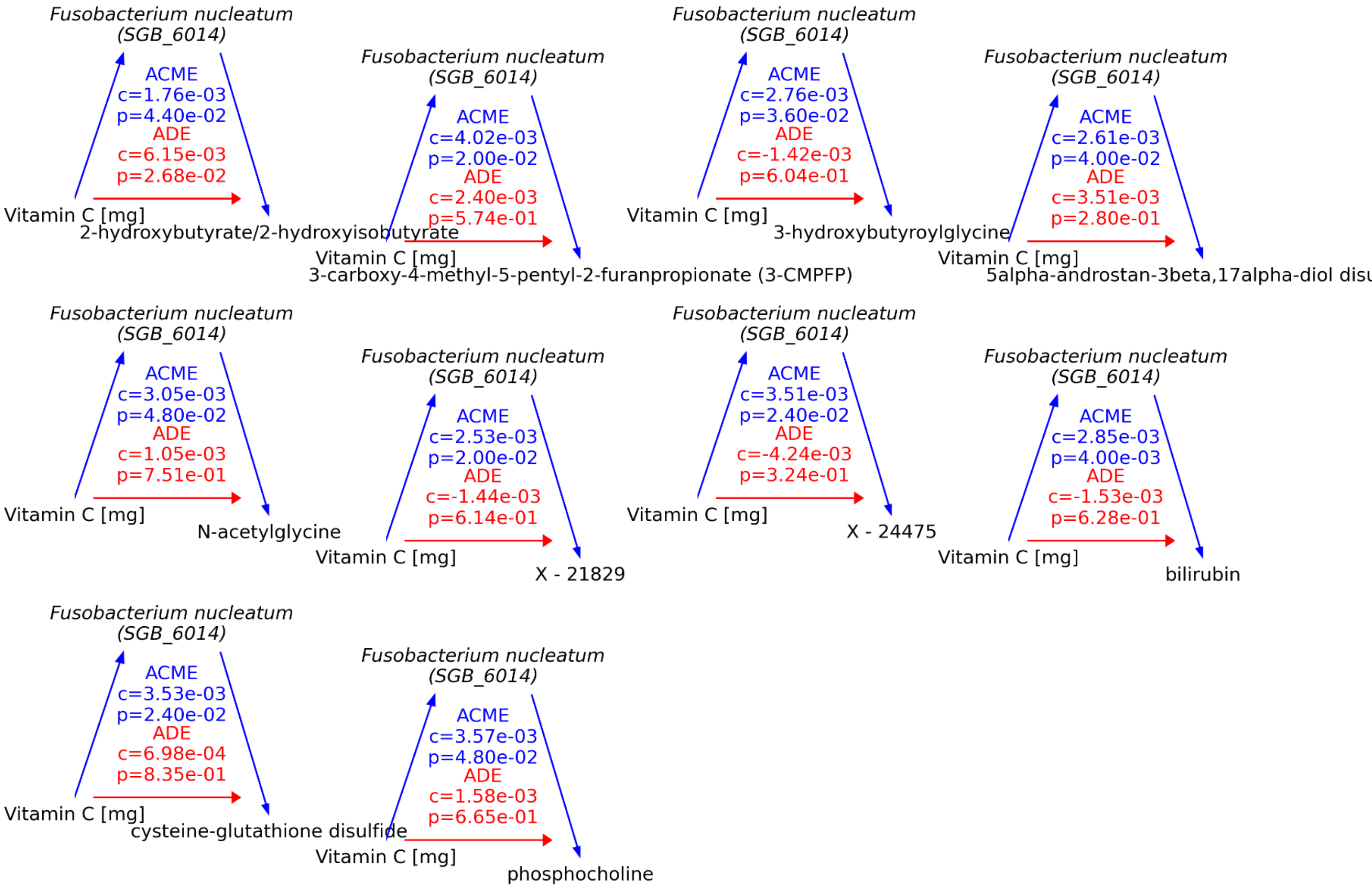

Figure S2. The microbiome mediates the diet's effect

Each plot shows a path from a dietary feature to an outcome that is mediated by the microbiome (two-sided bootstrap $p<0.05$). The left apex is the dietary feature, the right apex is the outcome and the upper apex is the microbial mediator. The title specifies the diet group ("PPT" or "MED" diet), type of microbial mediators (species, pathways or strains) and the type of outcome (glycemic measurements or metabolites and cytokines). The average direct effect (ADE) in red, the average causal mediated effect (ACME) in blue, c - effect size, p - p-value. Source data are provided as a Source Data file.

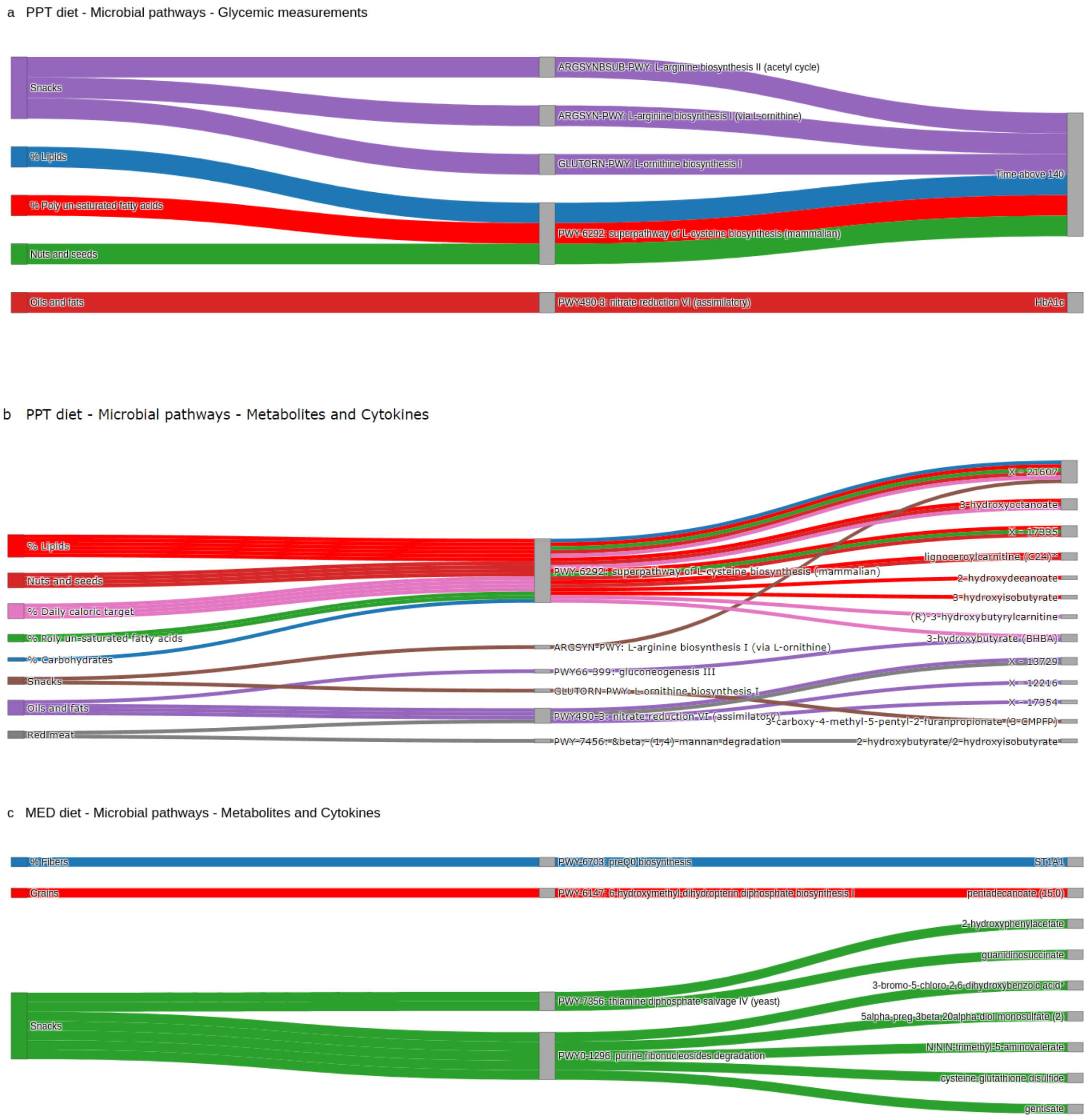

Figure S3. Microbial pathways mediate the diet's effect

Each alluvial plot shows paths from diet to outcomes that are mediated by oral and gut microbial pathways (two-sided bootstrap p<0.05). The outcomes in **a.** are glycemic measurements, and in **b.** and **c.** the outcomes are metabolites and cytokines. **a.** and **b.** show paths of the "PPT diet", while **c.** shows paths of the "MED diet". No mediating effect on glycemic measurements was found in the "MED diet". Source data are provided as a Source Data file.

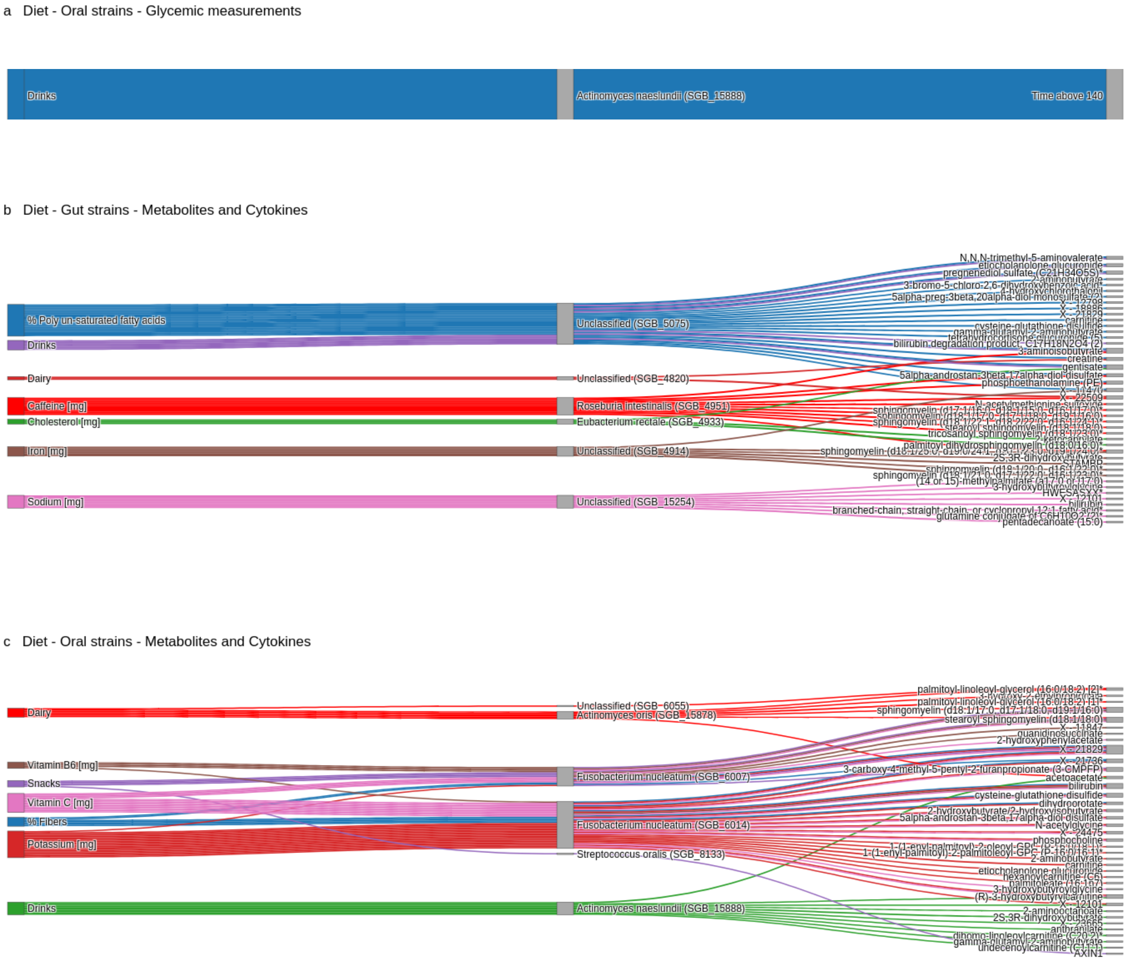

Figure S4. Microbial strains mediate the diet's effect

Each alluvial plot shows paths from diet to outcomes that are mediated by microbial strains (two-sided bootstrap $p<0.05$). The outcomes in **a.** are glycemic measurements, and in **b.** and **c.** the outcomes are metabolites and cytokines. **a.** and **c.** show paths of the oral environment, while **b.** shows paths of the gut environment. The “PPT diet” and “MED diet” were combined. No mediating effect on glycemic measurements was found in the gut environment. Source data are provided as a Source Data file.
