# Supplementary material for: Impact of dietary interventions on pre-diabetic oral and gut microbiome, metabolites and cytokines
Source: Nat Commun. 2023 Sep 4;14:5384. doi: 10.1038/s41467-023-41042-x (PMC10477304; doi:10.1038/s41467-023-41042-x)
Supplement: Supplementary file 3 — Description of Additional Supplementary Files [file 41467_2023_41042_MOESM3_ESM.pdf]

Description of Additional Supplementary Files

File Name: Supplementary Data 1
Description: Statistical tests

File Name: Supplementary Data 2
Description: Mediation analyses

File Name: Supplementary Data 3
Description: Metabolites prediction

File Name: Supplementary Data 4
Description: Microbial strains
