# Supplementary material for: Impact of dietary interventions on pre-diabetic oral and gut microbiome, metabolites and cytokines
Source: Nat Commun. 2023 Sep 4;14:5384. doi: 10.1038/s41467-023-41042-x (PMC10477304; doi:10.1038/s41467-023-41042-x)
Supplement: Supplementary file 8 — Reporting Summary [file 41467_2023_41042_MOESM8_ESM.pdf]

nature portfolio

Corresponding author(s): Saar Shoer

Last updated by author(s): Aug 8, 2023

Reporting Summary

Nature Portfolio wishes to improve the reproducibility of the work that we publish. This form provides structure for consistency and transparency in reporting. For further information on Nature Portfolio policies, see our Editorial Policies and the Editorial Policy Checklist.

Statistics

For all statistical analyses, confirm that the following items are present in the figure legend, table legend, main text, or Methods section.

n/a	Confirmed	
☐	☒	The exact sample size (*n*) for each experimental group/condition, given as a discrete number and unit of measurement
☐	☒	A statement on whether measurements were taken from distinct samples or whether the same sample was measured repeatedly
☐	☒	The statistical test(s) used AND whether they are one- or two-sided<br>*Only common tests should be described solely by name; describe more complex techniques in the Methods section.*
☐	☒	A description of all covariates tested
☐	☒	A description of any assumptions or corrections, such as tests of normality and adjustment for multiple comparisons
☐	☒	A full description of the statistical parameters including central tendency (e.g. means) or other basic estimates (e.g. regression coefficient) AND variation (e.g. standard deviation) or associated estimates of uncertainty (e.g. confidence intervals)
☐	☒	For null hypothesis testing, the test statistic (e.g. *F*, *t*, *r*) with confidence intervals, effect sizes, degrees of freedom and *P* value noted<br>*Give P values as exact values whenever suitable.*
☒	☐	For Bayesian analysis, information on the choice of priors and Markov chain Monte Carlo settings
☒	☐	For hierarchical and complex designs, identification of the appropriate level for tests and full reporting of outcomes
☐	☒	Estimates of effect sizes (e.g. Cohen's *d*, Pearson's *r*), indicating how they were calculated

Our web collection on statistics for biologists contains articles on many of the points above.

Software and code

Policy information about availability of computer code

Data collection	Dietary data was collected using a designated smartphone application called "Personalized Nutrition Project" (version 1).
Data analysis	Microbiome samples were processed using the following programs: URA0, Trimmomatic0.38, Bowtie2, HUMAnN3, DIAMOND2 and MetaPhlAn4. Computational analysis was performed in python (v3.7) using the following packages: numpy (v1.21.0) and pandas (v1.2.5) for data processing, scipy (v1.7.0), mne (v0.23.0), statannot (v0.2.3) and pingouin (v0.5.3) for statistical analyses, matplotlib (v3.4.3), seaborn (v0.12.0) and plotly (v4.5.4) for creating figures. The code is deposited in https://github.com/saarshoer/Pre-diabetes.git.

For manuscripts utilizing custom algorithms or software that are central to the research but not yet described in published literature, software must be made available to editors and reviewers. We strongly encourage code deposition in a community repository (e.g. GitHub). See the Nature Portfolio guidelines for submitting code & software for further information.

Data

Policy information about availability of data

All manuscripts must include a data availability statement. This statement should provide the following information, where applicable:

- Accession codes, unique identifiers, or web links for publicly available datasets
- A description of any restrictions on data availability
- For clinical datasets or third party data, please ensure that the statement adheres to our policy

The diet, microbiome, metabolites and cytokines data generated in this study are in the Source Data file provided with this paper as supplementary file 1. The raw microbiome samples generated in this study have been deposited in the European Nucleotide Archive (ENA) under accession code PRJEB64861. External datasets

used in this study are human genome 19, microbial genomes Pasolli et al., and microbial pathways MetaCyc24.

Human research participants

Policy information about studies involving human research participants and Sex and Gender in Research.

Reporting on sex and gender	Sex [%males] in PPT diet was 41% and in MED diet was 46%
Population characteristics	Age [years] at baseline in PPT diet was 50.37±7.86 and in MED diet was 50.92±8.03<br>Each diet group had 100 pre-diabeteic participants
Recruitment	Participants enrollment and recruitment occurred between January 2017 and January 2019. The date of final follow-up was March 2020. Participants included in the study met two glycemic criteria for pre-diabetes as defined by the 2010 American Diabetes Association guidelines: (1) GPF levels between 100 and 125 mg/dL (5.6 and 6.9 mmol/L) and (2) HbA1c level between 5.7 and 6.5% (39 and 48 mmol/mol). Other inclusion criteria were age of 18–65 years and capability to work with a smartphone app on a daily basis (for dietary intake logging). Key exclusion criteria were any use of diabetes or weight loss medications, use of antibiotics in the three months before enrollment, diagnosed chronic diseases, or chronic use of medications that affect glucose/energy metabolism or %HbA1c. The recruitment process relied primarily on self-assignment of volunteers from Israel who self-reportedly declared themselves as having pre-diabetes on the trial website. Registrants were screened for the above eligibility criteria based on a questionnaire and if qualified underwent a screening visit to determine final eligibility at the central medical laboratory of the trial (AMC Medical Center Laboratory, Ltd.). For more information please see Ben-Yacov et al.
Ethics oversight	Clinical trial registration number NCT03222791, http://www.clinicaltrials.gov. The study was approved by the Institutional Review Board (IRB) ethics committee of the Weizmann Institute of Science (reference number: 398-1) and by a sponsor-appointed data and safety monitoring board.

Note that full information on the approval of the study protocol must also be provided in the manuscript.

Field-specific reporting

Please select the one below that is the best fit for your research. If you are not sure, read the appropriate sections before making your selection.

☒ Life sciences ☐ Behavioural & social sciences ☐ Ecological, evolutionary & environmental sciences

For a reference copy of the document with all sections, see nature.com/documents/nr-reporting-summary-flat.pdf

Life sciences study design

All studies must disclose on these points even when the disclosure is negative.

Sample size	To estimate the required sample size, we performed power analysis while estimating effect size using the results of different studies describing controlled diet intervention aimed at reducing glycemic parameters. Our goal was to detect a difference of at least 0.1% in the reduction of HbA1C between control group and experimental group. Based on the study of Lindeberg S et al., the standard deviation of HbA1C reduction is 0.26 and the projected sample size with alpha of 0.05 and power of 0.8 is 106 for each group. Hence, with an estimated dropout rate of 10%, we planned to recruit 117 people for each arm totaling 234 individuals.
Data exclusions	Features were filtered out if they exist in less than 20 samples. This criteria was not pre-established.
Replication	Not applicable as this is a human clinical trial.
Randomization	Participants were randomly assigned in a 1:1 ratio to a PPT or MED diet. Covariate adaptive randomization with minimization was performed to ensure minimal differences between the groups in six prognostic baseline characteristics: age, sex, weight, BMI, HbA1c and fasting plasma glucose (FPG).
Blinding	Participants and measurers were blinded to arm assignment, while the investigators and dietitians were not.

Reporting for specific materials, systems and methods

We require information from authors about some types of materials, experimental systems and methods used in many studies. Here, indicate whether each material, system or method listed is relevant to your study. If you are not sure if a list item applies to your research, read the appropriate section before selecting a response.

Materials & experimental systems

n/a	Involved in the study
☒	☐ Antibodies
☒	☐ Eukaryotic cell lines
☒	☐ Palaeontology and archaeology
☒	☐ Animals and other organisms
☐	☒ Clinical data
☒	☐ Dual use research of concern

Methods

n/a	Involved in the study
☒	☐ ChIP-seq
☒	☐ Flow cytometry
☒	☐ MRI-based neuroimaging

Clinical data

Policy information about clinical studies

All manuscripts should comply with the ICMJEguidelines for publication of clinical research and a completedCONSORT checklist must be included with all submissions.

Clinical trial registration: Clinical trial registration number NCT03222791, http://www.clinicaltrials.gov.

Study protocol: Full trial protocol can be accessed in http://www.clinicaltrials.gov.

Data collection: Participants enrollment and recruitment occurred between January 2017 and January 2019. The date of final follow-up was March 2020. The trial's site was the Weizmann Institute of Science and the central medical laboratory of the trial was AMC Medical Center Laboratory, Ltd.

Outcomes: The primary and secondary outcomes of this clinical trial derived from blood tests and anthropometric measurements were previously reported, this manuscript is of an exploratory analysis, accordingly no pre-defined outcomes were set.
